# Social threat, fronto-cingulate-limbic morphometry, and symptom course in depressed adolescents: a longitudinal investigation

**DOI:** 10.1017/S0033291722002239

**Published:** 2022-09-19

**Authors:** Amar Ojha, Giana I. Teresi, George M. Slavich, Ian H. Gotlib, Tiffany C. Ho

**Affiliations:** 1 Center for Neuroscience, University of Pittsburgh, Pittsburgh, PA, USA;; 2 Center for Neural Basis of Cognition, University of Pittsburgh, Pittsburgh, PA, USA;; 3 Department of Psychology, University of Pittsburgh, Pittsburgh, PA, USA;; 4 Department of Psychiatry and Biobehavioral Sciences, University of California, Los Angeles, CA, USA;; 5 Department of Psychology, Stanford University, Stanford, CA, USA; 6 Department of Psychiatry and Behavioral Sciences, Weill Institute for Neurosciences, University of California, San Francisco, CA, USA

**Keywords:** Adolescence, amygdala, anterior cingulate cortex, depression, nucleus accumbens, social stress

## Abstract

**Background.:**

Psychosocial stressors characterized by social threat, such as interpersonal loss and social rejection, are associated with depression in adolescents. Few studies, however, have examined whether social threat affects fronto-cingulate-limbic systems implicated in adolescent depression.

**Methods.:**

We assessed lifetime stressor severity across several domains using the Stress and Adversity Inventory (STRAIN) in 57 depressed adolescents (16.15 ± 1.32 years, 34 females), and examined whether the severity of social threat and non-social threat stressors was associated with gray matter volumes (GMVs) in the anterior cingulate cortex (ACC), amygdala, hippocampus, and nucleus accumbens (NAcc). We also examined how lifetime social threat severity and GMVs in these regions related to depressive symptoms at baseline and over 9 months.

**Results.:**

General stressor severity was related to greater depression severity at baseline and over 9 months. Moreover, greater severity of social threat (but not non-social threat) stressors was associated with smaller bilateral amygdala and NAcc GMVs, and smaller bilateral surface areas of caudal and rostral ACC (all *p*_FDR_ ⩽ 0.048). However, neither social threat nor non-social threat stressor severity was related to hippocampal GMVs (all *p*_FDR_ ⩾ 0.318). All fronto-cingulate-limbic structures that were associated with the severity of social threat were negatively associated with greater depression severity over 9 months (all *p*_FDR_ ⩽ 0.014).Post-hoc analyses suggested that gray matter morphometry of bilateral amygdala, NAcc, and rostral and caudal ACC mediated the association between social threat and depression severity in adolescents over 9 months (all *p*_FDR_ < 0.048).

**Conclusions.:**

Social threat specifically affects fronto-cingulate-limbic pathways that contribute to the maintenance of depression in adolescents.

## Introduction

Depression is a leading cause of disability worldwide (World Health Organization, 2017), with nearly one in five people experiencing depression during their lifetime (Friedrich, 2017). The lifetime prevalence of depression is especially alarming in adolescents, with a cumulative incidence of 13.6% in males and 36.1% in females by age 18 (Breslau et al., 2017). Moreover, individuals who develop depression during adolescence, as compared to those who develop depression later in life, tend to experience more severe and recurrent episodes of the disorder (Naicker, Galambos, Zeng, Senthilselvan, & Colman, 2013; Zisook et al., 2007). Viewed in this context, adolescence represents an important developmental period marked by heightened vulnerability for the onset and adverse consequences of depression. Therefore, it is critical to identify psychosocial and neurobiological mechanisms that contribute to depression in adolescents.

### Social stressors and depression: the role of social threat

Although stressful life events often precede and increase risk for depressive episodes across the lifespan (Hammen, 2005, 2006), this effect is especially pronounced in adolescents (Lewinsohn, Allen, Seeley, & Gotlib, 1999; Monroe & Harkness, 2005). This finding is not surprising given that adolescence is a period during which environmental demands have outsized effects on neurodevelopment (Ho, 2019; Larsen & Luna, 2018), an effect that coincides with experiences of heightened stress. Importantly, exposure to psychosocial stressors characterized by social threat, including interpersonal loss and social rejection, has been found to be a strong proximal risk factor not only for the onset of depression in adolescence (Slavich, O’Donovan, Epel, & Kemeny, 2010a), but also for the persistence and recurrence of depressive symptoms over time (Hammen, 2006, 2009). Specifically, experiences of social threat have been found to uniquely predict adolescent depression (Flynn & Rudolph, 2011; Vrshek-Schallhorn et al., 2015) and to be associated with heightened risk for the recurrence of depression in this age group (Sheets & Craighead, 2014). Specific experiences of social threat that have been related to adolescent depression include peer rejection, bullying, victimization (Platt, Kadosh, & Lau, 2013), poor-quality relationships, and relationship dissolutions (Mirsu-Paun & Oliver, 2017).

In addition to being a proximal risk factor for depression, experiences of early adversity – and specifically of social threat during early life – are also associated with adolescent-onset depression (LeMoult et al., 2020). Exposure to early adversity shapes responses to stressors during adolescence and throughout the lifespan (Claessens et al., 2011). Therefore, it is important to consider cumulative experiences of stressors, including those that may be chronic and span different developmental periods, when investigating the impact of social threat on neural and behavioral outcomes. Few studies, however, have systematically assessed whether experiences of social threat are uniquely associated with neurophenotypes of depression in adolescents.

### Cortical neural correlates of social threat and adolescent depression

We also have an incomplete understanding of how lifetime social threat severity may alter specific neurobiological pathways that underlie adolescent depression. Because interpersonal relationships are particularly important in adolescence, adolescents may be more sensitive to social cues than are children and adults, possibly through relevant neurobiological pathways (Blakemore & Mills, 2014; Somerville, Jones, & Casey, 2010; Steinberg, 2005). Indeed, several cortical and subcortical structures have been implicated in social stressors and depression (Slavich & Irwin, 2014). The anterior cingulate cortex (ACC) – a broad region with multiple functions, including integrating sensory and emotional stimuli, computing value-based signals for action selection, and supporting effective conflict monitoring – is a critical neural substrate underlying the pathophysiology of adolescent depression (Botvinick, Cohen, & Carter, 2004; Lichenstein, Verstynen, & Forbes, 2016; Shenhav, Botvinick, & Cohen, 2013). Evidence from studies using structural and functional magnetic resonance imaging have consistently identified the rostral ACC (rACC) – a division of the ACC that receives a diversity of corticolimbic inputs and is involved in affective processing – as especially relevant to depression. Specifically, alterations in the morphology and function of the rACC have been associated with depressive phenotypes in youth (Boes, McCormick, Coryell, & Nopoulos, 2008; Ho et al., 2017a). Researchers have found that rACC thickness predicted treatment response to transcranial magnetic stimulation in patients with medication-refractory depression predicts treatment response to repetitive transcranial magnetic stimulation (Boes et al., 2018) as well as response to internet-delivered cognitive behavioral therapy (Webb et al., 2018). In addition, activation in the rACC to affective stimuli, as well as intrinsic functional connectivity between the rACC and other frontolimbic regions, have been shown to predict improvements in depression across psychosocial and pharmacological therapies in adolescents and young adults with depression (Davey, Cearns, Jamieson, & Harrison, 2021; Jamieson, Harrison, Razi, & Davey, 2022). Together, these studies suggest that characteristics of the rACC as central to the experience of depression and may be a useful biomarker of treatment response.

Another line of research suggests that the ACC influences how the body and brain respond to socially stressful events. Investigators have found that the ACC is more active in response to social exclusion than inclusion, and that increased activation is related to greater self-reported distress (Eisenberger, Lieberman, & Williams, 2003). Separate work has found greater activation in the dorsal ACC during social exclusion (*v*. inclusion) to be associated with increased inflammatory reactivity, implicating the ACC and related circuitry as a pathway through which social stress may contribute to depressive phenotypes (Slavich, Way, Eisenberger, & Taylor, 2010b). Increases in ACC activation also have been documented in response to social-evaluative threat (Eisenberger, Taylor, Gable, Hilmert, & Lieberman, 2007; Wager et al., 2009). Further, increases in rACC activation have been reported to mediate cardiovascular responses to social-evaluative threat; specifically, threat caused increased activation in the rACC, which, in turn, was associated with increased heart rate (Wager et al., 2009). Consistent with this work, in a recent review, activation in the ACC was the most common fMRI response to social exclusion in studies examining this phenomenon (Wang, Braun, & Enck, 2017). Finally, animal research corroborates human neuroimaging work: following a bilateral ACC lesion in rhesus monkeys, investigators observed a reduction in prosocial preferences (Basile, Schafroth, Karaskiewicz, Chang, & Murray, 2020), suggesting that ACC functioning is not only implicated in social processes, but may also be necessary for engagement in certain social behaviors.

### Subcortical neural correlates of social threat and adolescent depression

Several subcortical regions, including the hippocampus, amygdala, and nucleus accumbens (NAcc), also have been implicated in stress processing, affective and motivated behaviors, and depression (Ho, 2021; Ho et al., 2022; Tottenham & Galván, 2016). The hippocampus is involved in learning and memory, and, as a target of stress hormones, is a core structure underlying the neural regulation of affect (Sheline, Liston, & McEwen, 2019). Not surprisingly, depression is characterized in part by hippocampal dysfunction, with smaller hippocampal volumes being documented as among the most robust neurophenotypes of depression (Ho et al., 2022; Schmaal et al., 2017). In contrast, the amygdala detects salient emotional stimuli in the environment and, therefore, is highly sensitive to social-environmental stressors (Anderson & Phelps, 2001; Cunningham & Brosch, 2012; van Marle, Hermans, Qin, & Fernández, 2009). Alterations in amygdala function and structure are common both in patients with depression and in individuals with histories of life stressor exposure, especially during sensitive periods of development (Cohen et al., 2013; Frodl et al., 2002; Siegle, Steinhauer, Thase, Stenger, & Carter, 2002). Researchers have found volumetric alterations in the amygdala in the context of stress: whereas acute stress has been associated with greater basolateral amygdala spine density, chronic stress has been related to dendritic growth in this region in animal models (Roozendaal, McEwen, & Chattarji, 2009). Conversely, lower perceived stress, measured using a self-report questionnaire following a mindfulness-based stress reduction intervention, was associated with reductions in gray matter density in the right basolateral amygdala (Hölzel et al., 2010).

Finally, the NAcc – part of the ventral striatum – receives dopaminergic inputs from the ventral tegmental area and is a core component of reward-learning and motivated behavior circuitry (Knutson, Adams, Fong, & Hommer, 2001; Salamone, Correa, Mingote, & Weber, 2005). In depression, reductions in NAcc activation have been found to be associated with symptoms of anhedonia (Wacker, Dillon, & Pizzagalli, 2009); further, researchers have found smaller NAcc gray matter volume (GMV) in depressed individuals than in healthy controls (Zhang et al., 2021). Recent research has posited that life stress drives symptoms of anhedonia in depression via reward pathways involving the NAcc (Pizzagalli, 2014), which may be especially relevant for adolescent depression given the heightened sensitivity to reward during this developmental period (Auerbach, Admon, & Pizzagalli, 2014; Galván, 2010). Indeed, Lee et al. (2020) recently found that reduced NAcc volumes serve as an indirect pathway by which interpersonal stressors (e.g. peer conflict) lead to adolescent depression.

### Goals of the present study

The research described above details several cortical and subcortical structures that are affected by stress, that undergo significant maturation during adolescence, and that are implicated in depression during this important developmental period. Although converging evidence suggests that these regions are susceptible to the effects of social stressors, the extent to which the morphology of these regions is associated with the severity of depression in adolescents is not clear.

To address this gap in our knowledge, we investigated the extent to which gray matter morphometry of fronto-cingulate-limbic regions are susceptible to stressors characterized by social threat severity over the life course and, further, the extent to which these regions are related to the severity of depression in adolescents with depressive disorders. Fifty-seven depressed adolescents were assessed at baseline and provided up to five reports of depression severity over the course of 9 months. We examined how lifetime exposure to different types of social stressors assessed by the Stress and Adversity Inventory for Adolescents (Adolescent STRAIN; Slavich, Stewart, Esposito, Shields, & Auerbach, 2019) was related to the morphometry of cortical and subcortical brain structures. We focused on the perceived severity of stressors occurring over the lifespan given evidence that perceived stress severity affects neurobiological development above and beyond the number of life events experienced (Ho et al., 2017b; Ho & King, 2021). Consistent with prior research, we hypothesized that greater cumulative severity of (i.e. experiencing more severe) social threat over the life course would be associated with greater depressive symptom severity at baseline and over the 9-month study period. We also hypothesized that experiencing more lifetime social threat severity would be related to lower cortical thickness and surface areas of rostral and caudal anterior cingulate cortex (rACC, cACC, respectively) and to smaller hippocampal, amygdalar, and NAcc GMVs. To further interrogate these findings, we conducted specificity analyses to test for lateralization effects for all significant associations with lifetime social threat severity and, in addition, tested whether these findings were specific to lifetime social threat severity or were associated with non-social threat severity as well. Finally, we tested whether fronto-cingulate-limbic morphology sensitive to lifetime social threat severity mediated the association between lifetime social threat severity and depressive symptom severity longitudinally over 9 months.

## Methods

### Participants

Sixty-six depressed adolescents between 13 and 18 years old were recruited from the San Francisco Bay Area community as part of a longitudinal study examining neurobiological mechanisms underlying adolescent stress and depression (see Walker et al., 2020, for a description of the study protocol). In accord with the Declaration of Helsinki, all study components were approved by the Institutional Review Boards (IRB) at Stanford University and the University of California, San Francisco.

Inclusion criteria at the baseline visit of the study included fluency in English and the presence of current threshold or subthreshold major depressive disorder (MDD), dysthymia, or depressive disorder not otherwise specified via child or parent report according to DSM-IV criteria as assessed with the Kiddie Schedule for Affective Disorders and Schizophrenia – Present and Lifetime Version (K-SADS-PL; see Clinical assessments, below). See online Supplementary material for more information on inclusion/exclusion criteria. Participants were included in the present study if they completed the Adolescent STRAIN (see below) and provided usable structural MRI data (see below), resulting in a final sample of 57 participants.

### Procedure

The present study is part of an ongoing three-wave longitudinal study (Walker et al., 2020). Eligible participants (based on a phone screen) were invited to participate in a behavioral session (T1V1) in which adolescents and their parent/legal guardian completed diagnostic interviews and self-report questionnaires. Eligibility was confirmed following completion of the diagnostic interviews (see Clinical assessments, below); those who met criteria for study participation (see online Supplementary material) were invited to participate in the remainder of the study procedure, including an MRI scan (T1V2; session interval: 10.75 ± 5.83 days; see MRI scanning acquisition, below). Following the MRI scan, participants were invited to complete self-report questionnaires about their symptoms of depression every other month from home (e.g. M3, M5, M7, etc.) in addition to a behavioral follow-up assessment at 9 months (T2). For the present study, we included participants who provided follow-up data up to T2 (as of 1 July 2021), for longitudinal analyses for a maximum of five assessments per participant (for additional protocol details, see Walker et al., 2020). See online Supplementary Fig. S1 for details on the study timeline, sample sizes, and intervals per follow-up assessment for the present investigation.

### Clinical assessments

Trained research assistants administered the K-SADS-PL (Kaufman et al., 1997; Kaufman, Birmaher, Brent, Ryan, & Rao, 2000) and the Children’s Depressive Rating Scale – Revised (CDRS-R; Poznanski & Mokros, 1996) to adolescents and their parent/legal guardian at the initial behavioral session to assess eligibility.

### Lifetime stressor exposure

The Adolescent STRAIN is an online, interview-based system for assessing cumulative lifetime exposure to stressors in adolescents ages 10–18 years (Slavich et al., 2019; see https://www.strainsetup.com/). For the purposes of this study, we focused on the total severity of social threat that occurred across the life course. This variable was computed by summing the severity scores for all of the acute and chronic stressors endorsed that involved interpersonal loss or humiliation. To test the specificity of these effects, we also computed a non-social threat severity score by subtracting each participant’s lifetime social threat severity score from their total lifetime stressor severity score. The Adolescent STRAIN was administered to participants online within 3 weeks of their initial behavioral session (T1V1). The instrument has excellent psychometric properties and has been validated against psychological, behavioral, and clinical outcomes (Cazassa, Oliveira, Spahr, Shields, & Slavich, 2020; Slavich & Shields, 2018; Sturmbauer, Shields, Hetzel, Rohleder, & Slavich, 2019). Because our goal was to empirically test associations between stressor exposure and behavioral and neurobiological outcomes described in life stress models of depression, we focused on the total severity of social threat that occurred across the entire life course.

### Depression and anxiety symptom severity

Self-reported severity of depressive symptoms was assessed using the Reynolds Adolescent Depression Scale (RADS-2; Reynolds, 2010), a 30-item questionnaire designed to assess the severity of depressive symptoms in adolescents ages 11–20 years and that has been shown to have excellent validity and reliability (Reynolds, 2010). RADS-2 items are rated on a four-point Likert scale from 1 (*Almost never*) to 4 (*Most of the time*), with total scores ranging from 30 to 120. The RADS-2 was administered to participants at all baseline and follow-up assessments. Cronbach’s *α* was high at each assessment (T1 = 0.88, M3 = 0.91, M5 = 0.93, M7 = 0.91, T2 = 0.91; overall = 0.91).

Self-reported severity of anxiety symptoms was assessed using the Multidimensional Scale for Anxiety for Children (MASC-2; March, 2012), a 39-item questionnaire designed to assess severity of anxiety symptoms in youth ages 8–19 years and that has been shown to have excellent validity and reliability (March, Parker, Sullivan, Stallings, & Conners, 1997; Wei et al., 2014). MASC-2 items are rated on a four-point Likert scale from 0 (*Never true about me*) to 3 (*Often true about me*), with total scores ranging from 0 to 117. The MASC-2 was administered to participants at all baseline and follow-up assessments. Cronbach’s *α* was high at each assessment (T1 = 0.92, M3 = 0.92, M5 = 0.92, M7 = 0.93, T2 = 0.93; overall = 0.92).

### MRI scanning acquisition

All MRI scans were acquired at the Stanford Center for Cognitive and Neurobiological Imaging (CNI) with a 3 T MRI scanner (General Electric Healthcare Systems) and 32-channel head coil (Nova Medical Inc., Wilmington, MA, USA). Forty-six participants were scanned on a Discovery MR750 and 11 participants were scanned on the SIGNA Ultra High Performance after COVID-19 mitigation procedures were put into place. Therefore, in all statistical analyses, we also included session type (pre-COVID, post-COVID) as a dichotomous covariate.

### MRI quality control

**Fig. 1.**
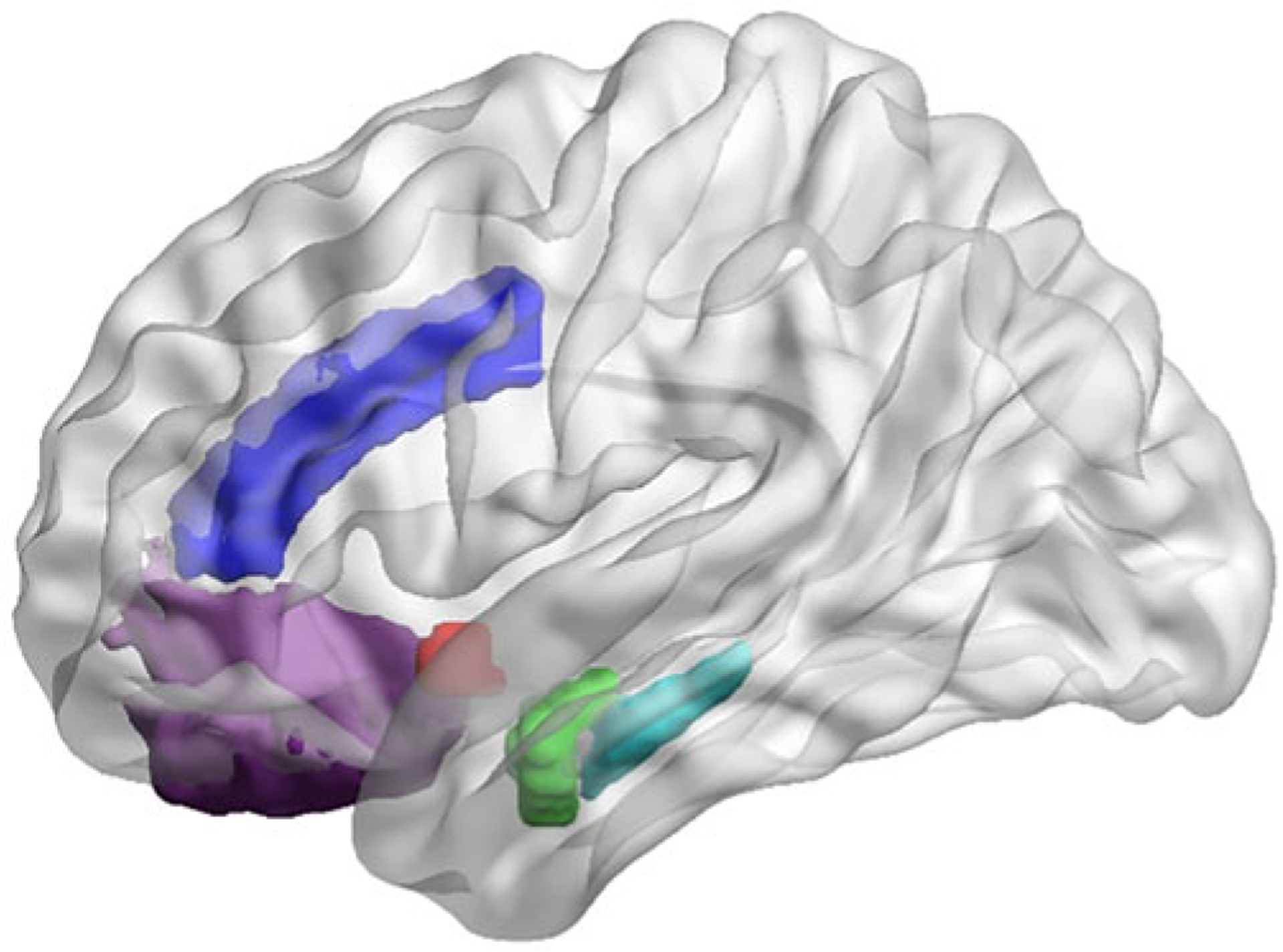
Illustration of cortical and subcortical ROIs in standard space (for visualization purposes): rostral anterior cingulate cortex (dark purple), caudal anterior cingulate cortex (dark blue); nucleus accumbens (red), amygdala (green), hippocampus (teal). Segmentation of and estimation of gray matter for each ROI was performed within each individual. ROI, region of interest.

MRI scans were visually inspected for motion artifacts before tissue segmentation and surface-based parcellation using FreeSurfer 6.0, as previously described (Ho et al., 2018, 2021). See Fig. 1 for representative segmentations of regions of interest. Bilateral GMVs for each structure were calculated by averaging across the two hemispheres. Given that we did not have hypotheses regarding laterality in the limbic structures of interest, we used bilateral regions of interest (ROIs) for these analyses. We followed up all analyses in which significant effects were identified for a given limbic ROI by conducting *post-hoc* analyses examining possible laterality effects (see next section for more details).

### Statistical analyses

#### Descriptive statistics and zero-order correlations among study variables

Correlations between the main study variables and covariates were tested using Pearson’s correlations. Student’s *t* tests and χ^2^ tests were used to determine if the nine participants excluded from final analyses differed from those included in our final analytic sample.

#### Social threat and depression severity

We used linear regressions to test whether lifetime social threat severity was associated with RADS-2 scores cross-sectionally, and linear mixed effects (LME) modeling to test whether higher levels of lifetime social threat severity assessed at baseline predicted greater depression severity over 9 months (Kuznetsova, Brockhoff, & Christensen, 2017). Covariates for these cross-sectional analyses included age and a dichotomous variable for antidepressant medication use at time of assessment, along with session type (for models that included morphometry metrics, as there was a hardware scanner upgrade post-COVID). All LME models included a subject-specific random intercept, a random slope based on time since baseline (T1V1) in years, and age at baseline, time-varying antidepressant medication use as a dichotomous variable, time since baseline in years, and a dichotomous variable for session type (where appropriate) as covariates. We also present results with sex as a covariate. Predictor and outcome variables were *z*-scored for all longitudinal statistical models.

#### Social threat and fronto-cingulate-limbic brain morphometry

We also used multiple linear regressions to investigate whether lifetime social threat severity was associated with subcortical GMV and cortical thickness and surface area for our regions of interest. Covariates for these cross-sectional analyses included age and a dichotomous variable for antidepressant medication use at time of assessment, along with session type (pre-COVID, post-COVID). We also present results with sex as an additional covariate. For all significant associations, in *post-hoc* analyses, we tested the two hemispheres separately to investigate whether there was lateralization specificity.

#### Specificity of the effects of social threat

To test the specificity of associations to lifetime social threat severity, we tested whether depression, as well as subcortical and cortical morphology, was associated with non-social threat severity.

#### Exploratory longitudinal mediation analyses

We conducted exploratory longitudinal analyses using fixed-effects modeling (for the path between lifetime social threat severity and brain morphometry metrics) and LMEs (for the paths predicting RADS-2 scores) to test whether fronto-cingulate-limbic structures statistically mediated the association between lifetime social threat severity and depressive symptom severity over 9 months. Specifically, for each brain metric, we used Monte Carlo simulations to estimate the 95% confidence intervals (CI) of the indirect effect of GMV on the association between lifetime social threat severity and RADS-2 scores.

#### Specificity of the effects to depression

To test whether these patterns were specific to depression, we also tested whether lifetime social threat and non-social threat severity, as well as subcortical and cortical morphology, were associated with anxiety as assessed with MASC-2 scores.

#### Exploratory whole-brain analyses

Although we took a hypothesis-driven approach to the present study, we also conducted a whole-brain surface-based voxel-wise analysis to complement our primary analyses and test whether other cortical regions were associated with lifetime social threat severity. See online Supplementary material for methodological details.

#### Statistical approach

We report the fixed-effect parameters of all cross-sectional and longitudinal statistical models (Lüdecke, Ben-Shachar, Patil, & Makowski, 2020). Given concerns about degrees of freedom, we included sex assigned at birth as an additional covariate in *post-hoc* sensitivity analyses. All reported *β* values are standardized. Finally, each of our statistical models met assumptions for linear modeling (i.e. normality of residuals, homoscedasticity). Significance was set at *p* < 0.05 with false discovery rate (FDR) correction (*α* = 0.05). All statistical analyses were conducted in R v. 4.0.3 (R Core Team, 2020). See online Supplementary material for methodological details.

## Results

### Descriptive statistics and zero-order correlations among study variables

Demographic and clinical characteristics at baseline for the 57 participants are presented in Table 1. These participants did not differ on any demographic or clinical characteristics from the nine participants who were excluded due to missing data (all *p*s > 0.15). A correlation matrix of cross-sectional associations between the main variables of interest is presented in online Supplementary Fig. S2.

### Social threat and depression severity

**Fig. 2.**
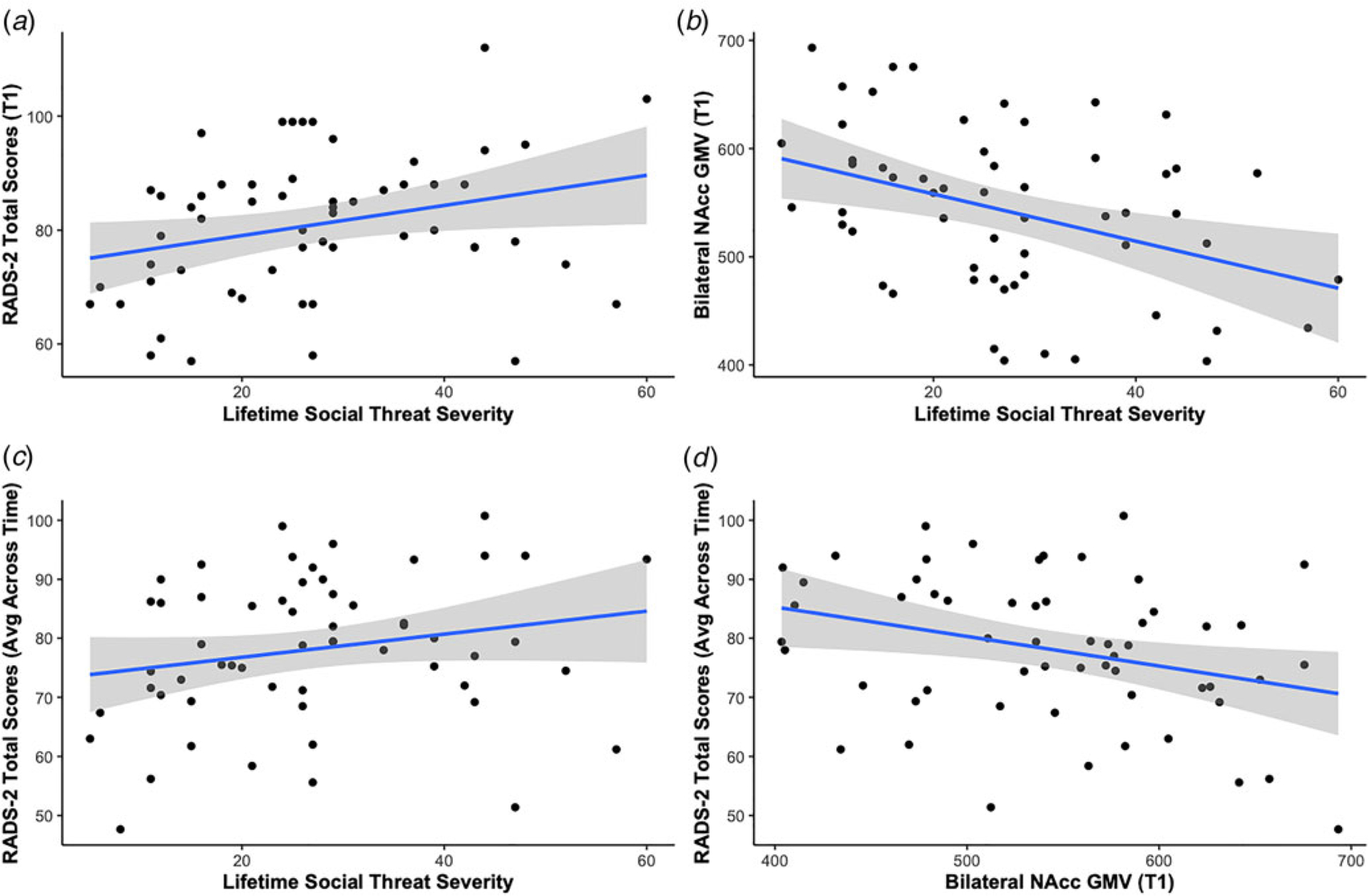
Summary of primary results. Scatterplot and fitted regression line illustrating the association between (a) lifetime social threat severity and depression severity, (b) lifetime social threat severity and gray matter volumes of bilateral NAcc, (c) lifetime social threat severity and depression severity over the course of the study (intraindividual average RADS-2 score across all timepoints), and (d) gray matter volumes of bilateral NAcc and depression severity over the course of the study (intraindividual average RADS-2 score across all timepoints). NAcc, nucleus accumbens; RADS-2, Reynolds Adolescent Depression Scale, 2nd Edition; T1, baseline.

Higher reported lifetime social threat severity was associated with greater depression severity both cross-sectionally at baseline (*β* = 0.37, *p*_FDR_ = 0.005) and longitudinally over 9 months (*β* = 0.28, *p*_FDR_ = 0.027). See Table 2 and Fig. 2 for more details. These results were significant at baseline when including sex as a covariate (*β* = 0.35, *p* = 0.015, uncorrected) but not longitudinally (*β* = 0.22, *p* = 0.065, uncorrected). See online Supplementary Tables S1A and S1B. Including sex as a covariate did not significantly improve model fit. See online Supplementary Tables S2A and S2B.

### Social threat and fronto-cingulate-limbic brain morphometry

Higher reported lifetime social threat severity was associated with smaller bilateral amygdala (*β* = −0.39, *p*_FDR_ = 0.020) and NAcc GMVs (*β* = −0.35, *p*_FDR_ = 0.020) but not with hippocampal GMV (*β* = −0.15, *p*_FDR_ = 0.318). Lifetime social threat severity was not associated with either rACC or cACC cortical thickness (all *p*_FDR_ > 0.475). In contrast, higher reported social threat severity was associated with smaller cACC and rACC surface areas (cACC: *β* = −0.34, *p*_FDR_ = 0.048; rACC: *β* = −0.33, *p*_FDR_ = 0.048). See Tables 3 and 4.

When including sex as a covariate, higher reported lifetime social threat remained significantly associated with both smaller amygdala and NAcc GMVs (amygdala: *β* = −0.29, *p* = 0.038, uncorrected; NAcc: *β* = −0.30, *p* = 0.036, uncorrected); however, neither rACC nor cACC surface area was associated with lifetime social threat severity after covarying for sex (all *ps* ⩾ 0.074, uncorrected). See online Supplementary Table S1C. Including sex as a covariate significantly improved model fit for associations between lifetime social threat severity and amygdala GMV, rACC surface area, and cACC surface area. See online Supplementary Table S2C.

### Specificity of effects to social threat

We tested whether the associations described above were specific to lifetime social threat severity. As hypothesized, greater self-reported non-social threat severity (total lifetime stressor severity – total lifetime social threat severity) was associated with greater depression severity cross-sectionally (*β* = 0.45, *p*_FDR_ = 0.002) and over 9 months (*β* = 0.27, *p*_FDR_ = 0.027). See Table 2. Including sex as a covariate did not change the significance of the effect of non-social threat severity on RADS-2 at baseline (*p* = 0.005, uncorrected) but did attenuate the significance of the effect on RADS-2 longitudinally (*p* = 0.062, uncorrected). A summary of these analyses can be found in online Supplementary Tables S1A and S1B. Including sex as a covariate did not improve model fit. See online Supplementary Tables S2A and S2B. Conversely, as hypothesized, lifetime non-social threat severity was not associated with hippocampal, amygdalar, or NAcc GMV (all *p*_FDR_ ⩾ 0.218) or with cACC or rACC surface areas or cortical thickness (all *p*_FDR_ ⩾ 0.120), indicating that social threat severity specifically affects gray matter morphometry of these regions. Including sex as a covariate did not change the lack of a significant association for any of these effects (all *p*s ⩾ 0.109). See online Supplementary Table S1D. Including sex as a covariate significantly improved model fit for associations between lifetime non-social threat severity and amygdala GMV, cACC surface area, and rACC surface area. See online Supplementary Table S2D.

### Exploratory longitudinal mediation analyses

We conducted exploratory longitudinal analyses to examine whether the subcortical and cortical regions that were significantly associated with lifetime social threat severity also predicted depressive symptom severity over 9 months. More specifically, we constructed LME models testing the associations between baseline rACC and cACC surface areas, NAcc and amygdala GMVs, and depression symptoms over time. All structures were negatively associated with depression symptoms over 9 months (all *β*s ⩽ −0.28, all *p*_FDR_ < 0.014). See Table 5 for details. After including sex as an additional covariate, each of these structures were still significantly associated with depression symptoms at baseline (all *p*s < 0.018, uncorrected), with only NAcc GMVs and cACC surface areas associated with depression symptoms longitudinally (NAcc: *β* = −0.24, *p* = 0.037, uncorrected; cACC: *β* = −0.23, *p* = 0.045, uncorrected). See online Supplementary Tables S1E and S1F. Including sex as a covariate significantly improved model fit for associations between amygdala GMV, cACC surface area, and rACC surface area and RADS-2 at baseline but did not improve model fit for any of these models with RADS-2 longitudinally. See online Supplementary Tables S2B and S2C.

**Fig. 3.**
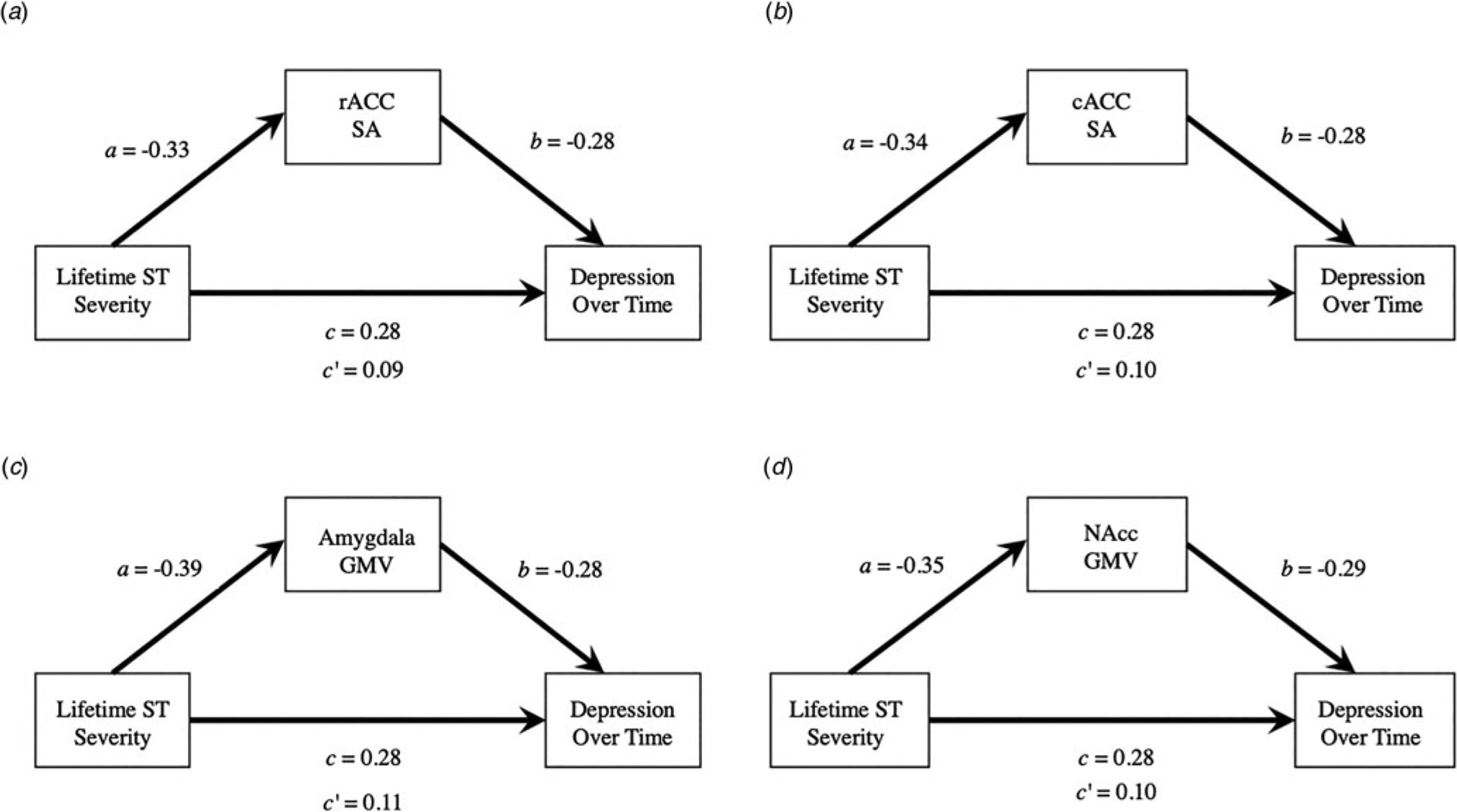
Summary of exploratory mediation analyses. Mediation model schematic illustrating associations between lifetime social threat severity, depression severity over the course of the study (intraindividual average RADS-2 score across all timepoints), and the indirect effect of (a) rACC SA, (b) cACC SA, (c) amygdala GMV, and (d) NAcc GMV. rACC, rostral anterior cingulate cortex; cACC, caudal anterior cingulate cortex; NAcc, nucleus accumbens; SA, surface area; GMV, gray matter volume; ST, lifetime social threat severity.

As a follow-up to these longitudinal analyses, we conducted *post-hoc* analyses to test whether the regions that demonstrated significant associations with lifetime social threat severity – bilateral baseline amygdala GMV, cACC surface area, NAcc GMV, rACC surface area – statistically mediated the association between lifetime social threat severity and depression symptom severity over 9 months. All of the indirect effects tested were significant [amygdala: *β* = 0.11, 95% CI (0.02–0.25); cACC: *β* = 0.10, 95% CI (0.008–0.23); NAcc: *β* = 0.10, 95% CI (0.01–0.23); rACC: *β* = 0.09, 95% CI (0.006–0.217)]. See Fig. 3 for more details.

### Specificity of effects to depression

Next, we tested whether the associations described above were specific to depression severity by examining associations with self-reported anxiety (MASC-2). We found that the effect of lifetime social threat severity on MASC-2 scores at baseline and over 9 months was similar to that of RADS-2 (baseline: *β* = 0.43, *p* = 0.002, uncorrected; longitudinal: *β* = 0.37, *p* = 0.002, uncorrected) and, in addition, that these effects were specific to lifetime social threat severity in that all associations with non-social threat severity were non-significant (*p*s > 0.214). See online Supplementary Tables S3A and S3B.

Amygdala and NAcc GMVs were significantly correlated with MASC-2 scores at baseline (amygdala: *β*s = −0.27, *p* = 0.033, uncorrected; NAcc: *β* = −0.39, *p* = 0.002, uncorrected). See online Supplementary Table S3C. Similarly, amygdala and NAcc GMVs, as well as cACC surface area, were significantly correlated with MASC-2 scores over 9 months (all *p*s < 0.044, uncorrected). See online Supplementary Table S3D for more details.

In addition, we found that only NAcc and amygdala GMVs statistically mediated the association between lifetime social threat severity and MASC-2 scores over 9 months. See online Supplementary Table S3E for more details.

### Exploratory whole-brain analyses

Finally, we conducted exploratory whole-brain analyses to further interrogate these data. We found that greater lifetime social threat severity was associated with lower cortical surface areas in the left fusiform gyrus, left inferior parietal region, left postcentral gyrus, and right superior frontal surface area, lower cortical volumes in the left fusiform gyrus, and left inferior parietal region. Consistent with our original analyses, cortical thickness was not associated with lifetime social threat severity. For more details, see online Supplementary Table S4 and Fig. S3.

## Discussion

Although separate lines of evidence have implicated exposure to major life stressors in the onset and maintenance of depression in adolescents, it is unclear how various *types* of stressors occurring over the life course affect specific aspects of neural morphology to influence the pathophysiology of adolescent depression. We investigated this issue in the present study by examining how the perceived severity of social stressors occurring over the lifespan was associated with gray matter morphometry in the ACC, amygdala, hippocampus, and NAcc. We also investigated whether the lifetime social threat severity and GMVs in these fronto-cingulate-limbic regions were associated with severity of depression symptoms at baseline and their persistence over 9 months. As hypothesized, greater lifetime social threat severity was associated with more depression severity at baseline and over the course of 9 months. Also consistent with our hypotheses, greater lifetime social threat severity was associated with smaller bilateral amygdala and NAcc GMV. Surprisingly, neither lifetime social nor non-social threat severity was related to hippocampal GMV.

Lifetime social threat severity was not related to cACC or rACC cortical thickness but higher reported lifetime social threat severity was associated with smaller bilateral surface areas in both subregions. As hypothesized, NAcc and amygdala GMVs and cACC and rACC surface areas were all associated with lifetime social threat severity but were not related to non-social threat severity, suggesting that these regions are differentially sensitive to social threat severity. Finally, all fronto-cingulate-limbic structures that were associated with lifetime social threat severity were also related to severity of depression over the course of 9 months.

These findings are generally consistent with prior cross-sectional research examining associations among NAcc GMV, social stressors, and depression in adolescents. For example, a recent cross-sectional study of 78 depressed adolescents and 47 healthy controls found that NAcc GMV mediated the association between peer problems (e.g. victimization and/or bullying) – one common form of social stress experienced during adolescence – and adolescent depression (Lee et al., 2020). Consistent with our findings regarding the specificity of the NAcc in linking social stressors to depression in adolescents, Lee et al. did not find such associations with amygdala or hippocampal GMVs. Notably, however, they reported *larger* NAcc GMV in the depressed adolescent group, which they interpreted as reflecting heightened sensitivity to adverse peer relationships. These discrepant findings between our study and Lee et al.’s investigation may be explained in part by the fact that Lee et al. operationalized ‘peer problems’ as the sum of peer victimization and bullying behaviors, obscuring both whether these findings were driven by the victims or the aggressors of adverse peer relationships, and the role of NAcc in the context of social stress and adolescent depression.

Although we are unable to make mechanistic inferences due to the correlational nature of our study, it is possible that the observation of smaller NAcc GMV as a function of lifetime social threat severity may also be due to the fact that our sample (mean age: 16.15 years) was older than Lee et al.’s sample (mean age: 14 years), suggesting that there is a loss in NAcc GMV experienced across adolescence that may reflect the downstream effects of stressor exposure, including inflammatory processes, on brain-derived neurotropic factors and neuronal integrity (Felger and Treadway, 2017) (for a more detailed overview of these processes, see Walker et al., 2020). Although they did not examine social threat severity specifically, Auerbach and colleagues (2022) recently found smaller NAcc GMVs in adolescents with depression/anxiety relative to healthy controls and, furthermore, found that smaller NAcc GMVs predicted depressive symptoms over 6 months. Similarly, in a study using the Adolescent Brain Cognitive Development (ABCD) study dataset (*N* = 11 876), investigators compared subcortical GMVs in 9- and 10-year-old children who were at high or low risk for depression based on parental history of depression and found that high-risk children had smaller right putamen and NAcc GMVs than did low-risk children, which was driven primarily by the effects of a maternal history of depression on striatal structures in the right hemisphere (Pagliaccio, Alqueza, Marsh, & Auerbach, 2020). In addition, in a prospective longitudinal study, smaller NAcc GMVs in girls ages 12–16 years predicted a diagnosis of MDD by age 18 (Whittle et al., 2014). Although we are unable to definitively demonstrate that stressor exposure leads to insufficient growth or sustained neuronal loss (as observed by reductions in gray matter morphometry as measured by MRI) as opposed to delayed development, our findings, while viewed in the context of these other studies, suggest that smaller NAcc volumes are already present prior to the onset of a depressive episode possibly due to stressor exposure, and that these smaller NAcc volumes persist throughout adolescence and may contribute to the maintenance of depression as well as, potentially, anxiety disorders.

Other studies have also reported associations between depression and gray matter in mesial temporal structures, with more nuanced associations between stress severity and these structures. For instance, Rosso et al. (2005) reported smaller bilateral amygdalar GMVs in children with depression compared to healthy controls but no differences in hippocampal volumes. Similar to Pagliaccio et al.’s (2020) findings described above, Saleh et al. (2012) found in an adult cohort (18–65 years of age) that familial risk for depression was associated with smaller amygdalar GMVs. The effect of adolescent depression on the hippocampus, however, remains inconsistent. Several researchers have found smaller hippocampal GMVs (and hippocampal subfields) in adolescents with depression as compared to healthy controls (e.g. MacMaster et al., 2008; MacMaster & Kusumakar, 2004; Whittle et al., 2014), as well as in adults with adolescent-onset depression (Ho et al., 2022); however, others have not found these differences (e.g. Pannekoek et al., 2014; Rosso et al., 2005). In our study, lifetime stressor severity was not associated with hippocampal GMVs in depressed adolescents. It may be that the *timing* of stress affects hippocampal growth; indeed, there is evidence that stress exposure during childhood (prior to age 6) has a disproportionate impact on adolescent hippocampal volumes (Humphreys et al., 2019). As recent reviews have highlighted (Ho & King, 2021; Slavich, 2019; Teicher, Samson, Anderson, & Ohashi, 2016), stressor exposure timing as well as stressor type need to be considered when attempting to elucidate the effects by which life stressor exposure influences brain regions implicated in adolescent depression.

With respect to cortical structures, human neuroimaging research has demonstrated that ACC morphology, and specifically smaller surface area of the ACC, is a robust neurophenotype of adolescent depression (e.g. Pannekoek et al., 2014; Schmaal et al., 2017). Although smaller rACC volumes have been found to be associated with depressive symptoms, including in subclinical children (e.g. Boes et al., 2008), most research has not considered cortical morphology in the context of specific types of stress (e.g. social threat) in adolescent depression. Our study advances this area of research by demonstrating that lifetime social threat severity in depressed adolescents is associated with smaller surface area in rACC and cACC. Moreover, our findings that rACC and cACC surface areas are significant mediators of associations between stressors and depression symptoms – but not anxiety symptoms – suggest there may be specificity with respect to cortical structures representing a pathway by which experiences of social threat severity lead to depression. Broadly, our findings are also consistent with previous studies demonstrating that morphological characteristics of the ACC represent biomarkers of depression that are predictive of treatment response (Boes et al., 2018; Davey et al., 2021; Jamieson et al., 2022; Webb et al., 2018).

Although rACC volumes have been found to be negatively associated with depression, including in children with subclinical symptoms (e.g. Boes et al., 2008), most research has not considered finer-grained aspects of cortical morphology in the context of adolescent stress and depression. This is critical, given that changes in volumes could be due to changes in cortical thickness or in surface area, or both. Whereas cortical thickness likely reflects dendritic arborization and pruning and has been shown to decrease linearly with age, surface area reflects cortical folding and gyrification and has a curvilinear association with age, with maximum area during adolescence and young adulthood (Storsve et al., 2014; Wierenga, Langen, Oranje, & Durston, 2014). Interestingly, cortical thickness and cortical surface area have been found to have distinct genetic bases (Panizzon et al., 2009), with the latter area exhibiting greater genetic heritability as well as stronger effects from early environmental factors (Fjell et al., 2019). Researchers have speculated that lower surface area could therefore reflect pre-existing risk for depression in youth (Schmaal 2019). Smaller ACC surface area specifically appears to be a neurophenotype of adolescent depression (e.g. Pannekoek et al., 2014; Schmaal et al., 2017) but it is unclear if or how stressors may influence this. Our study advances this area of research by demonstrating that lifetime social threat severity in depressed adolescents is associated with smaller surface area in rACC and cACC.

Our findings should be interpreted in light of several study limitations. First, the sample size was modest, which may have limited our statistical power to find significant effects that we observed as non-significant. Second, given the disruption in in-person data collection due to the COVID-19 pandemic, we were only able to examine brain morphometry at a single time-point, thereby limiting our ability to make inferences regarding the extent to which life stressors influence neurodevelopment of these structures and their sustained contributions to the severity and persistence of depressive symptoms. This is especially relevant for understanding the role of the NAcc as a potential mediator of the association between social stressors and depression, given evidence that the NAcc (as well as other striatal structures) continues to mature throughout adolescence (e.g. Ernst et al., 2005; Herting et al., 2018; Parr et al., 2021) and is especially sensitive to social stimuli (both positive and negative) during this period of development (Foulkes & Blakemore, 2016). Third, for ethical reasons, we did not manipulate exposure to life stressors in these participants; consequently, the findings are correlational in nature, preventing us from drawing causal inferences. Indeed, subcortical regions (e.g. NAcc, amygdala) may already be smaller in individuals at familial risk for depression (e.g. Pagliaccio et al., 2020; Saleh et al., 2012). It is noteworthy, however, that studies using animal models suggest causal relations among stress, these neural structures, outcomes related to depression and anxiety (e.g. Francis et al., 2015; Heshmati et al., 2020). Fourth, the heterogeneity of depressive symptoms in adolescents (Lamers et al., 2012) may reflect biological heterogeneity in fronto-cingulate-limbic circuitry across subtypes of depression (Buch & Liston, 2021); future studies should examine large, diverse samples from which multimodal (e.g. neurobiological and behavioral) longitudinal data can be obtained to explore factors related to stress, brain development, and the onset and course of depression. Finally, although not studied in this sample, we wish to highlight the potential role of social support in potentially mitigating the negative effects of stress on depressive symptoms in adolescents (e.g. Rueger, Malecki, Pyun, Aycock, & Coyle, 2016; van Harmelen et al., 2016, 2017, 2021). To examine these effects, we encourage future studies to investigate the extent to which levels of social support – and which sources of social support – might attenuate the association between lifetime social stressor severity and depressive symptoms in adolescents.

Despite these limitations, the present study has several strengths. First, the longitudinal design of the study enabled us to investigate how lifetime stressor exposure predicted depression severity over the course of nine months and, in addition, how these associations were mediated by gray matter morphometry of stress-sensitive brain regions. Second, we assessed stressors that occurred over the entire life course, which has rarely been done (Slavich, 2019). Finally, our findings add to a growing literature indicating that associations between life stressors and neural correlates of adolescent depression differ depending on the specific types of stressors experienced (e.g. Andersen & Teicher, 2008; Ho & King, 2021; Sheridan & McLaughlin, 2020; Slavich & Irwin, 2014).

Looking forward, it will be important for future research to focus on understanding how social stressors specifically contribute to the onset, course, and maintenance of adolescent depression. Although non-social threat severity was also associated with depression severity in our sample, this form of stress severity was not associated with any of the brain regions we investigated, suggesting that different neurobiological circuitry or processes are relevant for encoding various forms of stressors. Delineating relations between life stressors and depression in adolescence is particularly important given that this is not only a developmental period of increased risk for MDD, but also one when lifelong risk for this burdensome disorder can still be effectively mitigated (Slavich & Sacher, 2019; Twenge, Cooper, Joiner, Duffy, & Binau, 2019). That said, depression is a clinically and biologically heterogeneous disorder that differs in its biological etiology and response to treatments, likely based in part on key facets of life stressors experienced preceding a depressive episode (Ho & King, 2021). A critical future research direction will be to examine other critical aspects of stress exposures (e.g. acute *v*. chronic stressors, abuse *v*. neglect, frequency *v*. count of stressors) and to test the extent to which these stressors have differential neurobiological impacts based on the developmental stage of the individual.

In sum, the present findings elucidate how lifetime social threat severity is related to fronto-cingulate-limbic morphometry in a well-characterized sample of depressed adolescents. Consistent with emerging work in this area, the social threat severity-sensitive regions we identified may represent promising biomarkers associated with the severity and possibly the persistence of depression in adolescents (as well as with anxiety, a common comorbidity with depression). Studies aimed at characterizing mechanisms by which different types of life stressors alter neurodevelopment and explain clinical heterogeneity are promising strategies needed to understand MDD and inform the development of novel treatments and interventions for this prevalent and costly disorder.

## Supplementary Material

Supplement

## Figures and Tables

**Table 1.** Descriptive statistics of participant demographic, clinical, and neural characteristics for the 57 participants with Adolescent STRAIN and structural fMRI data at baseline

Variable	Descriptive statistics
*Demographic characteristics*
Age at V1 (years)	16.15 ± 1.32 (13.65–18.37)
Time between V1 and V2 (days)	10.54 ± 5.71 (1–29)
Sex (female/male)	34 (59.65%)/23 (40.35%)
Gender
Male	22 (38.60%)
Female	29 (50.88%)
Non-binary/other	6 (10.53%)
Sexual orientation
Straight/heterosexual	25 (43.86%)
LGBTQ+	25 (43.86%)
Prefer not to say/missing	7 (12.28%)
Ethnicity
Hispanic/Latinx	12 (21.05%)
Not Hispanic/Latinx	45 (78.95%)
Race
White	27 (47.37%)
Black/African American	2 (3.51%)
American Indian/Alaska Native	2 (3.51%)
Asian	11 (19.30%)
Native Hawaiian/Pacific Islander	0 (0%)
Multiracial	10 (17.54%)
Other	5 (8.77%)
Highest parental education
Less than a high school diploma	0 (0%)
High school graduate or equivalent	1 (1.75%)
Some college (no degree)	6 (10.53%)
Associate degree	2 (3.51%)
Bachelor’s degree	17 (29.82%)
Master’s degree	19 (33.33%)
Doctoral or professional degree	9 (15.79%)
Unknown/missing	3 (5.26%)
Annual household income
Less than $20000	3 (5.26%)
$20 000-$34 999	1 (1.75%)
$35 000-$49 999	1 (1.75%)
$50 000-$74 999	4 (7.02%)
$75 000-$99 999	3 (5.26%)
Over $100 000	41 (71.93%)
Unknown/missing	4 (7.02%)
*Clinical characteristics*
RADS-2 total	80.95 ± 12.45 (57–112)
CDRS-R total	47.56 ± 11.9 (26–81)
Age of current depressive episode onset	13.76 ± 2.26 (4–17)
Number of depressive episodes	1.82 ± 1.4 (1–9)
Current psychotropic medication	27 (47.37%)
Antidepressant	19 (33.33%)
Antipsychotic	3 (5.26%)
Stimulant	5 (8.77%)
Benzodiazepine	1 (1.75%)
Other^a^	10 (17.54%)
Concurrent therapy	18 (31.58%)
Therapy^b^	29 (50.88%)
Lifetime comorbidity^c^
Anxiety disorders	29 (50.88%)
Obsessive compulsive disorder	3 (5.26%)
Eating disorders	4 (7.02%)
Disruptive, impulse control, and conduct disorders	4 (7.02%)
Post-traumatic stress disorder	9 (15.79%)
Attention-deficit/hyperactivity disorder	13 (22.81%)
Other	1 (1.75%)
Unknown/missing	4 (7.18%)
*Neural characteristics*
ICV (divided by 1000)	1537.94 ± 162.45 (1210–1860) [3]
Bilateral amygdala GMV	1700.64 ± 173.69 (1316.35–2070.95) [3]
R amygdala GMV	1784.68 ± 177.20 (1427.4–2185.4) [3]
L amygdala GMV	1616.59 ± 191.58 (1205.3–2105.2) [3]
Bilateral hippocampus GMV	4198.24 ±408.05 (3168.75–5038.70) [3]
R hippocampus GMV	4297.16 ± 426.50 (3453.7–5363.0) [3]
L hippocampus GMV	4099.31 ± 439.95 (2381.6–5011.1) [3]
Bilateral NAcc GMV	541.78 ± 75.09 (403.45–693.25) [3]
R NAcc GMV	555.92 ± 76.07 (392.1–714.0) [3]
L NAcc GMV	527.65 ± 88.46 (348.7–746.5) [3]
Bilateral caudal ACC cortical thickness	2.82 ± 0.21 (2.29–3.38) [3]
R caudal ACC thickness	2.73 ± 0.23 (2.22–3.49) [3]
L caudal ACC thickness	2.92 ± 0.24 (2.37–3.55) [3]
Bilateral caudal ACC surface area	683.25 ± 103.40 (516–932) [3]
R caudal ACC surface area	746.89 ± 154.32 (538–1121) [3]
L caudal ACC surface area	619.62 ± 116.12 (415–1061) [3]
Bilateral rostral ACC cortical thickness	3.05 ± 0.16 (2.69–3.46) [3]
R rostral ACC thickness	3.05 ± 0.24 (2.65–3.74) [3]
L rostral ACC thickness	3.04 ± 0.17 (2.73–3.61) [3]
Bilateral rostral ACC surface area	734.96 ± 114.69 (528.0–976.5) [3]
R rostral ACC surface area	619.54 ± 120.48 (415–910) [3]
L rostral ACC surface area	850.38 ± 135.44 (590–1160) [3]

*Note*: All continuous values are reported as mean ± s.d. (min–max). Numbers in brackets [ ] indicate the number of missing or unusable responses. Categorical variables are reported as percentage (count). Lifetime comorbidities (includes past and current reports integrated across parent and child interviews) are reported as percentage (count). Abbreviations: V1, visit 1; V2, visit 2; LGBTQ+, lesbian, gay, bisexual, transgender, queer; RADS-2, Reynolds Adolescent Depression Scale, 2nd Edition; CDRS-R, Children’s Depression Rating Scale–Revised; ICV, intracranial volume; R, right; L, left; GMV, gray matter volume; NAcc, nucleus accumbens; ACC, anterior cingulate cortex.

a Other medications taken by participants, with () indicating count, include gabapentin (1), lamotrigine (3), trazodone (4), prazosin (1), buspar (1), dextromethorphan (1), and cannabidiol (1).

b Therapy indicates the percentage (count) of participants who reported attending therapy sessions for their depression in the 2 months prior to their first visit.

c Lifetime comorbidity disorder groupings, with () indicating count for individual disorders: *anxiety disorders*: panic disorder (3), social phobia (12), simple phobia (5), agoraphobia (2), generalized anxiety disorder (21); *eating disorders*: anorexia nervosa (3), bulimia nervosa (0), eating disorder not otherwise specified (3); *disruptive, impulse control, and conduct disorders*: oppositional defiant disorder (2), conduct disorder (2); *other disorders*: autism spectrum disorder (1), disruptive mood dysregulation disorder (1).

**Table 2.** Fixed effects estimated from linear models testing for associations between social and non-social threat severity with depression symptoms at baseline (cross-sectional) and across nine months (longitudinal)

	*β*	s.e.	95% CI	*t* (df)	Uncorrected *p*	*p*_FDR_
Cross-sectional						
Lifetime social threat severity	0.37	0.13	[0.12–0.62]	2.94 (56)	0.005**	0.005**
Lifetime non-social threat severity	0.45	0.13	[0.20–0.71]	3.56 (56)	<0.001***	0.002**
Longitudinal						
Lifetime social threat severity	0.28	0.11	[0.06–0.50]	2.46 (53.23)	0.017*	0.027*
Lifetime non-social threat severity	0.27	0.12	[0.04–0.50]	2.27 (54.74)	0.027*	0.027*

For the longitudinal model, fixed-effects are estimated from a linear mixed-effects model that includes random intercepts and slopes. *β* refers to the standardized partial regression coefficients. s.e., standard error; CI, confidence interval; FDR, false discovery rate.

* *p* < 0.05,

** *p* < 0.01,

*** *p* < 0.001.

**Table 3.** Fixed effects estimated from linear models testing associations between social threat severity with subcortical gray matter volumes at baseline

	*β*	s.e. (*β*)	95% CI	*t* (df)	Uncorrected *p*	*p*_FDR_
Bilateral						
Amygdala	−0.39	0.14	[−0.67 to −0.11]	−2.79 (52)	0.007**	0.020*
Hippocampus	−0.15	0.15	[−0.44 to 0.15]	−1.01 (52)	0.318	0.318
NAcc	−0.35	0.14	[−0.63 to −0.08]	−2.57 (52)	0.013*	0.020*
Lateralized						
Right amygdala	−0.39	0.14	[−0.67 to −0.11]	−2.77 (52)	0.008**	0.016*
Left amygdala	−0.35	0.14	[−0.63 to −0.06]	−2.46 (52)	0.017*	0.023*
Right NAcc	−0.47	0.13	[−0.73 to −0.20]	−3.56 (52)	<0.001***	0.003**
Left NAcc	−0.20	0.14	[−0.49 to 0.09]	−1.39 (52)	0.172	0.172

*Post-hoc* lateralization analyses were only performed for structures exhibiting a significant bilateral effect. *β* refers to the standardized partial regression coefficients. CI, confidence interval; FDR, false discovery rate; NAcc, nucleus accumbens; s.e., standard error.

* *p* < 0.05,

** *p* < 0.01,

*** *p* < 0.001.

**Table 4.** Fixed effects estimated from linear models testing associations between social threat severity with cortical thickness and surface area at baseline

	*β*	s.e. (*β*)	95% CI	*t* (df)	Uncorrected *p*	*p*_FDR_
Bilateral						
rACC surface area	−0.33	0.14	[−0.61 to −0.05]	−2.33 (52)	0.024*	0.048*
rACC cortical thickness	0.11	0.15	[−0.19 to 0.40]	0.72 (52)	0.475	0.475
cACC surface area	−0.34	0.14	[−0.63 to −0.06]	−2.40 (52)	0.020*	0.048*
cACC cortical thickness	0.13	0.14	[−0.15 to 0.42]	0.92 (52)	0.360	0.475
Lateralized						
Right rACC surface area	−0.33	0.14	[−0.61 to −0.04]	−2.29 (52)	0.026*	0.082^+^
Left rACC surface area	−0.26	0.14	[−0.55 to 0.02]	−1.88 (52)	0.066^+^	0.088^+^
Right cACC surface area	−0.30	0.14	[−0.59 to −0.01]	−2.10 (52)	0.041*	0.082^+^
Left cACC surface area	−0.21	0.15	[−0.50 to 0.08]	−1.44	0.155	0.155

*Post-hoc* lateralization analyses were only performed for structures exhibiting a significant bilateral effect. *β* refers to the standardized partial regression coefficients. cACC, caudal anterior cingulate cortex; CI, confidence interval; FDR, false discovery rate; rACC, rostral anterior cingulate cortex; s.e., standard error.

* *p* < 0.05,

** *p* < 0.01,

*** *p* < 0.001.

**Table 5.** Fixed effects estimated from linear mixed-effects models testing associations between rACC surface area, cACC surface area, NAcc GMV, and amygdala GMV with depressive symptoms (RADS-2 scores) longitudinally over 9 months

	*β*	s.e. (*β*)	95% CI	*t* (df)	Uncorrected *p*	*p*_FDR_
Bilateral						
rACC surface area	−0.28	0.11	[−0.49 to −0.06]	−2.56 (51.08)	0.014*	0.014*
cACC surface area	−0.28	0.11	[−0.49 to −0.07]	−2.63 (52.21)	0.011*	0.014*
NAcc GMV	−0.29	0.11	[−0.51 to −0.07]	−2.58 (54.63)	0.013*	0.014*
Amygdala GMV	−0.28	0.11	[−0.50 to −0.07]	−2.60 (54.23)	0.012*	0.014*
Lateralized						
Right rACC	−0.33	0.10	[−0.53 to −0.13]	−3.17 (50.67)	0.003**	0.024*
Left rACC	−0.18	0.11	[−0.40 to 0.05]	−1.54 (50.93)	0.130	0.149
Right cACC	−0.26	0.11	[−0.47 to −0.05]	−2.38 (52.78)	0.021*	0.042*
Left cACC	−0.16	0.11	[−0.38 to 0.06]	−1.41 (51.08)	0.166	0.166
Right NAcc	−0.22	0.11	[−0.44 to 0.00]	−1.95 (54.66)	0.056^+^	0.075
Left NAcc	−0.29	0.11	[−0.51 to −0.08]	−2.66 (53.15)	0.010*	0.027*
Right amygdala	−0.22	0.11	[−0.44 to −0.01]	−2.03 (52.35)	0.047*	0.075
Left amygdala	−0.30	0.11	[−0.51 to −0.09]	−2.77 (55.30)	0.008**	0.027*

*Post-hoc* lateralization analyses were only performed for structures exhibiting a significant bilateral effect. *β* refers to the standardized partial regression coefficients. cACC, caudal anterior cingulate cortex; CI, confidence interval; FDR, false discovery rate; NAcc, nucleus accumbens; rACC, rostral anterior cingulate cortex; s.e., standard error.

* *p* < 0.05,

** *p* < 0.01,

*** *p* < 0.001.

## References

[R1] Andersen SL, & Teicher MH (2008). Stress, sensitive periods and maturational events in adolescent depression. Trends in Neurosciences, 31(4), 183–191. doi: 10.1016/j.tins.2008.01.004 18329735

[R2] Anderson AK, & Phelps EA (2001). Lesions of the human amygdala impair enhanced perception of emotionally salient events. Nature, 411, 305–309. doi: 10.1038/35077083 11357132

[R3] Auerbach RP, Admon R, & Pizzagalli DA (2014). Adolescent depression: Stress and reward dysfunction. Harvard Review of Psychiatry, 22(3), 139–148. doi: 10.1097/HRP.0000000000000034 24704785 PMC4016104

[R4] Auerbach RP, Pagliaccio D, Hubbard NA, Frosch I, Kremens R, Cosby E … Pizzagalli DA (2022). Reward-related neural circuitry in depressed and anxious adolescents: a human connectome project. Journal of the American Academy of Child and Adolescent Psychiatry, 61(2), 308–320. 33965516 10.1016/j.jaac.2021.04.014 PMC8643367

[R5] Basile BM, Schafroth JL, Karaskiewicz CL, Chang SW, & Murray EA (2020). The anterior cingulate cortex is necessary for forming prosocial preferences from vicarious reinforcement in monkeys. PLoS Biology, 18(6), e3000677. doi: 0.1371/journal.pbio.3000677 32530910 10.1371/journal.pbio.3000677 PMC7292358

[R6] Blakemore SJ, & Mills KL (2014). Is adolescence a sensitive period for sociocultural processing? Annual Review of Psychology, 65, 187–207. doi: 10.1146/annurev-psych-010213-115202 24016274

[R7] Boes AD, McCormick LM, Coryell WH, & Nopoulos P (2008). Rostral anterior cingulate cortex volume correlates with depressed mood in normal healthy children. Biological Psychiatry, 63(4), 391–397. doi: 10.1016/j.biopsych.2007.07.018 17916329 PMC2265665

[R8] Boes AD, Uitermarkt BD, Albazron FM, Lan MJ, Liston C, Pascual-Leone A, … Fox MD (2018). Rostral anterior cingulate cortex is a structural correlate of repetitive TMS treatment response in depression. Brain Stimulation, 11(3), 575–581. doi: 10.1016/j.brs.2018.01.029 29454551 PMC6136654

[R9] Botvinick MM, Cohen JD, & Carter CS (2004). Conflict monitoring and anteriorcingulate cortex: an update. Trends in Cognitive Sciences, 8(12), 539–546. 15556023 10.1016/j.tics.2004.10.003

[R10] Breslau J, Gilman SE, Stein BD, Ruder T, Gmelin T, & Miller E (2017). Sex differences in recent first-onset depression in an epidemiological sample of adolescents. Translational Psychiatry, 7(5), e1139. doi: 10.1038/tp.2017.105 28556831 PMC5534940

[R11] Buch AM, & Liston C (2021). Dissecting diagnostic heterogeneity in depression by integrating neuroimaging and genetics. Neuropsychopharmacology, 46 (1), 156–175. 32781460 10.1038/s41386-020-00789-3 PMC7688954

[R12] Cazassa MJ, Oliveira MDS, Spahr CM, Shields GS, & Slavich GM (2020). The Stress and Adversity Inventory for Adults (Adult STRAIN) in Brazilian Portuguese: Initial validation and links with executive function, sleep, and mental and physical health. Frontiers in Psychology, 10, 3083. doi: 10.3389/fpsyg.2019.03083. 32063871 PMC6999460

[R13] Claessens SE, Daskalakis NP, van Der Veen R, Oitzl MS, de Kloet ER, & Champagne DL (2011). Development of individual differences in stress responsiveness: an overview of factors mediating the outcome of early life experiences. Psychopharmacology, 214(1), 141–154. 21165737 10.1007/s00213-010-2118-y PMC3045508

[R14] Cohen MM, Jing D, Yang RR, Tottenham N, Lee FS, & Casey BJ (2013). Early-life stress has persistent effects on amygdala function and development in mice and humans. Proceedings of the National Academy of Sciences of the USA, 110(45), 18274–18278. doi: 10.1073/pnas.1310163110 24145410 PMC3831447

[R15] Cunningham WA, & Brosch T (2012). Motivational salience: Amygdala tuning from traits, needs, values, and goals. Current Directions in Psychological Science, 21(1), 54–59. doi: 10.1177/0963721411430832

[R16] Davey CG, Cearns M, Jamieson A, & Harrison BJ (2021). Suppressed activity of the rostral anterior cingulate cortex as a biomarker for depression remission. Psychological Medicine, 1–8. doi: 10.1017/S0033291721004323 PMC10123826 36762975

[R17] Eisenberger NI, Lieberman MD, & Williams KD (2003). Does rejection hurt? An fMRI study of social exclusion. Science, 302(5643), 290–292. doi:10.1126/science.1089134 14551436

[R18] Eisenberger NI, Taylor SE, Gable SL, Hilmert CJ, & Lieberman MD (2007). Neural pathways link social support to attenuated neuroendocrine stress responses. NeuroImage, 35(4), 1601–1612. doi: 10.1016/j.neuroimage.2007.01.038 17395493 PMC2710966

[R19] Ernst M, Nelson EE, Jazbec S, McClure EB, Monk CS, Leibenluft E, … Pine DS (2005). Amygdala and nucleus accumbens in responses to receipt and omission of gains in adults and adolescents. NeuroImage, 25 (4), 1279–1291. doi: 10.1016/j.neuroimage.2004.12.038 15850746

[R20] Felger JC, & Treadway MT (2017). Inflammation effects on motivation and motor activity: Role of dopamine. Neuropsychopharmacology, 42(1), 216–241. doi: 10.1038/npp.2016.143 27480574 PMC5143486

[R21] Fjell AM, Chen CH, Sederevicius D, Sneve MH, Grydeland H, Krogsrud SK … Walhovd KB (2019). Continuity and discontinuity in human cortical development and change from embryonic stages to old age. Cerebral Cortex, 29(9), 3879–3890. 30357317 10.1093/cercor/bhy266

[R22] Flynn M, & Rudolph KD (2011). Stress generation and adolescent depression: Contribution of interpersonal stress responses. Journal of Abnormal Child Psychology, 39(8), 1187–1198. doi: 10.1007/s10802-011-9527-1 21647600 PMC3199357

[R23] Foulkes L, & Blakemore SJ (2016). Is there heightened sensitivity to social reward in adolescence? Current Opinion in Neurobiology, 40, 81–85. doi: 10.1016/j.conb.2016.06.016 27420376

[R24] Francis TC, Chandra R, Friend DM, Finkel E, Dayrit G, Miranda J, … Lobo MK (2015). Nucleus accumbens medium spiny neuron subtypes mediate depression-related outcomes to social defeat stress. Biological Psychiatry, 77(3), 212–222. doi: 10.1016/j.biopsych.2014.07.021 25173629 PMC5534173

[R25] Friedrich MJ (2017). Depression is the leading cause of disability around the world. JAMA, 317(15), 1517. doi: 10.1001/jama.2017.3826 28418490

[R26] Frodl T, Meisenzahl E, Zetzsche T, Bottlender R, Born C, Groll C, … Möller HJ (2002). Enlargement of the amygdala in patients with a first episode of major depression. Biological Psychiatry, 51(9), 708–714. doi: 10.1016/s0006-3223(01)01359-2 11983184

[R27] Galván A (2010). Adolescent development of the reward system. Frontiers in Human Neuroscience, 4, 6. doi: 10.3389/neuro.09.006.2010 20179786 PMC2826184

[R28] Hammen C (2005). Stress and depression. Annual Review of Clinical Psychology, 1, 293–319. doi: 10.1146/annurev.clinpsy.1.102803.143938 17716090

[R29] Hammen C (2006). Stress generation in depression: Reflections on origins, research, and future directions. Journal of Clinical Psychology, 62(9), 1065–1082. doi: 10.1002/jclp.20293 16810666

[R30] Hammen C (2009). Adolescent depression: Stressful interpersonal contexts and risk for recurrence. Current Directions in Psychological Science, 18(4), 200–204. doi: 10.1111/j.1467-8721.2009.01636.x 20161119 PMC2741024

[R31] Herting MM, Johnson C, Mills KL, Vijayakumar N, Dennison M, Liu C, … Tamnes CK (2018). Development of subcortical volumes across adolescence in males and females: A multisample study of longitudinal changes. NeuroImage, 172, 194–205. doi: 10.1016/j.neuroimage.2018.01.020 29353072 PMC5910239

[R32] Heshmati M, Christoffel DJ, LeClair K, Cathomas F, Golden SA, Aleyasin H, … Russo SJ (2020). Depression and social defeat stress are associated with inhibitory synaptic changes in the nucleus accumbens. Journal of Neuroscience, 40(32), 6228–6233. doi: 10.1523/JNEUROSCI.2568-19.2020 32561672 PMC7406280

[R33] Ho TC (2019). Stress and neurodevelopment in adolescent depression. Biological Psychiatry, 86(10), e33–e35. doi: 10.1016/j.biopsych.2019.09.012 31648684 PMC7594880

[R34] Ho TC (2021). Editorial: Toward neurobiological-based treatments of depression and anxiety: A potential case for the nucleus accumbens. Journal of the American Academy of Child and Adolescent Psychiatry, S0890–8567(21), 00417–2. doi: 10.1016/j.jaac.2021.06.013 34216777

[R35] Ho TC, Cichocki AC, Gifuni AJ, Catalina Camacho M, Ordaz SJ, Singh MK, & Gotlib IH (2018). Reduced dorsal striatal gray matter volume predicts implicit suicidal ideation in adolescents. Social Cognitive and Affective Neuroscience, 13(11), 1215–1224. doi: 10.1093/scan/nsy089 30256980 PMC6234322

[R36] Ho TC, Gutman B, Pozzi E, Grabe HJ, Hosten N, Wittfeld K, … Schmaal L (2022). Subcortical shape alterations in major depressive disorder: Findings from the ENIGMA major depressive disorder working group. Human Brain Mapping, 43(1), 341–351. doi: 10.1002/hbm.24988 32198905 PMC8675412

[R37] Ho TC, & King LS (2021). Mechanisms of neuroplasticity linking adversity to depression: Developmental considerations. Translational Psychiatry, 11(1), 517. doi: 10.1038/s41398-021-01639-6 34628465 PMC8501358

[R38] Ho TC, King LS, Leong JK, Colich NL, Humphreys KL, Ordaz SJ, & Gotlib IH (2017b). Effects of sensitivity to life stress on uncinate fasciculus segments in early adolescence. Social Cognitive and Affective Neuroscience, 12(9), 1460–1469. doi: 10.1093/scan/nsx065 28460088 PMC5629927

[R39] Ho TC, Sacchet MD, Connolly CG, Margulies DS, Tymofiyeva O, Paulus MP, … Yang TT (2017a). Inflexible functional connectivity of the dorsal anterior cingulate cortex in adolescent major depressive disorder. Neuropsychopharmacology, 42(12), 2434–2445. doi: 10.1038/npp.2017.103 28553837 PMC5645733

[R40] Ho TC, Teresi GI, Ojha A, Walker JC, Kirshenbaum JS, Singh MK, & Gotlib IH (2021). Smaller caudate gray matter volume is associated with greater implicit suicidal ideation in depressed adolescents. Journal of Affective Disorders, 278, 650–657. doi: 10.1016/j.jad.2020.09.046 33039875 PMC9386733

[R41] Hölzel BK, Carmody J, Evans KC, Hoge EA, Dusek JA, Morgan L, … Lazar SW (2010). Stress reduction correlates with structural changes in the amygdala. Social Cognitive and Affective Neuroscience, 5(1), 11–17. doi: 10.1093/scan/nsp034 19776221 PMC2840837

[R42] Humphreys KL, King LS, Sacchet MD, Camacho MC, Colich NL, Ordaz SJ, … Gotlib IH (2019). Evidence for a sensitive period in the effects of early life stress on hippocampal volume. Developmental Science, 22(3), e12775. doi: 10.1111/desc.12775 30471167 PMC6469988

[R43] Jamieson AJ, Harrison BJ, Razi A, & Davey CG (2022). Rostral anterior cingulate network effective connectivity in depressed adolescents and associations with treatment response in a randomized controlled trial. Neuropsychopharmacology, 47(6), 1240–1248. doi: 10.1038/s41386-021-01214-z 34782701 PMC9018815

[R44] Kaufman J, Birmaher B, Brent D, Rao UMA, Flynn C, Moreci P, … Ryan N (1997). Schedule for affective disorders and schizophrenia for school-age children-present and lifetime version (K-SADS-PL): Initial reliability and validity data. Journal of the American Academy of Child & Adolescent Psychiatry, 36(7), 980–988. doi: 10.1097/00004583-199707000-00021 9204677

[R45] Kaufman J, Birmaher B, Brent DA, Ryan ND, & Rao U (2000). K-SADS-PL. Journal of the American Academy of Child & Adolescent Psychiatry, 39(10), 1208. doi: 10.1097/00004583-200010000-00002 11026169

[R46] Knutson B, Adams CM, Fong GW, & Hommer D (2001). Anticipation of increasing monetary reward selectivity recruits nucleus accumbens. Journal of Neuroscience, 21(16), RC159. doi: 10.1523/JNEUROSCI.21-16-j0002.2001 11459880 PMC6763187

[R47] Kuznetsova A, Brockhoff PB, & Christensen RH (2017). lmerTest package: Tests in linear mixed effects models. Journal of Statistical Software, 82 (1), 1–26. doi: 10.18637/jss.v082.i13

[R48] Lamers F, Burstein M, He JP, Avenevoli S, Angst J, & Merikangas KR (2012). Structure of major depressive disorder in adolescents and adults in the US general population. The British Journal of Psychiatry, 201(2), 143–150. doi: 10.1192/bjp.bp.111.098079 22700082 PMC3409428

[R49] Larsen B, & Luna B (2018). Adolescence as a neurobiological critical period for the development of higher-order cognition. Neuroscience & Biobehavioral Reviews, 94, 179–195. doi: 10.1016/j.neubiorev.2018.09.005 30201220 PMC6526538

[R50] Lee KH, Yoo JH, Lee J, Kim SH, Han JY, Hong SB, … Brent DA (2020). The indirect effect of peer problems on adolescent depression through nucleus accumbens volume alteration. Scientific Reports, 10(1), 1–9. doi: 10.1038/s41598-020-69769-3 32733056 PMC7392894

[R51] Lemoult J, Humphreys KL, Tracy A, Hoffmeister JA, Ip E, & Gotlib IH (2020). Meta-analysis: Exposure to early life stress and risk for depression in childhood and adolescence. Journal of the American Academy of Child and Adolescent Psychiatry, 59(7), 842–855. doi: 10.1016/j.jaac.2019.10.011. 31676392 PMC11826385

[R52] Lewinsohn PM, Allen NB, Seeley JR, & Gotlib IH (1999). First onset versus recurrence of depression: Differential processes of psychosocial risk. Journal of Abnormal Psychology, 108(3), 483–489. doi: 10.1037//0021-843x.108.3.483 10466272

[R53] Lichenstein SD, Verstynen T, & Forbes EE (2016). Adolescent brain development and depression: A case for the importance of connectivity of the anterior cingulate cortex. Neuroscience & Biobehavioral Reviews, 70, 271–287. doi: 10.1016/j.neubiorev.2016.07.024 27461914 PMC5074859

[R54] Lüdecke D, Ben-Shachar MS, Patil I, & Makowski D (2020). Extracting, computing and exploring the parameters of statistical models using R. Journal of Open Source Software, 5(53), 2445. doi: 10.21105/joss.02445

[R55] MacMaster FP, & Kusumakar V (2004). Hippocampal volume in early onset depression. BMC Medicine, 2(2), 1–6. doi: 10.1186/1741-7015-2-2 14969587 PMC333436

[R56] MacMaster FP, Mirza Y, Szeszko PR, Kmiecik LE, Easter PC, Taormina SP, … Rosenberg DR (2008). Amygdala and hippocampal volumes in familial early onset major depressive disorder. Biological Psychiatry, 63(4), 385–390. doi: 10.1016/j.biopsych.2007.05.005 17640621 PMC2268763

[R57] March JS (2012). Multidimensional anxiety scale for children (2nd Edn.). North Tonawanda, NY: Multi-Health Systems.

[R58] March JS, Parker JDA, Sullivan K, Stallings P, & Conners CK (1997). The multidimensional anxiety scale for children (MASC): Factor structure, reliability, and validity. Journal of the American Academy of Child & Adolescent Psychiatry, 36, 554–565. doi: 10.1097/00004583-199704000-00019 9100431

[R59] Mirsu-Paun A, & Oliver JA (2017). How much does love really hurt? A meta-analysis of the association between romantic relationship quality, breakups and mental health outcomes in adolescents and young adults. Journal of Relationships Research, 8, e5. doi: 10.1017/jrr.2017.6

[R60] Monroe SM, & Harkness KL (2005). Life stress, the ‘kindling’ hypothesis, and the recurrence of depression: Considerations from a life stress perspective. Psychological Review, 112(2), 417–445. doi: 10.1037/0033-295X.112.2.417 15783292

[R61] Naicker K, Galambos NL, Zeng Y, Senthilselvan A, & Colman I (2013). Social, demographic, and health outcomes in the 10 years following adolescent depression. Journal of Adolescent Health, 52(5), 533–538. doi: 10.1016/j.jadohealth.2012.12.016 23499382

[R62] Pagliaccio D, Alqueza KL, Marsh R, & Auerbach RP (2020). Brain volume abnormalities in youth at high risk for depression: Adolescent brain and cognitive development study. Journal of the American Academy of Child & Adolescent Psychiatry, 59(10), 1178–1188. doi: 10.1016/j.jaac.2019.09.032 31634568 PMC7165045

[R63] Panizzon MS, Fennema-Notestine C, Eyler LT, Jernigan TL, Prom-Wormley E, Neale M, … Kremen WS (2009). Distinct genetic influences on cortical surface area and cortical thickness. Cerebral Cortex, 19(11), 2728–2735. doi: 10.1093/cercor/bhp026 19299253 PMC2758684

[R64] Pannekoek JN, van der Werff SJ, van den Bulk BG, van Lang ND, Rombouts SA, van Buchem MA, … van der Wee NJ (2014). Reduced anterior cingulate gray matter volume in treatment-naive clinically depressed adolescents. NeuroImage: Clinical, 4, 336–342. doi: 10.1016/j.nicl.2014.01.007 24501702 PMC3913835

[R65] Parr AC, Calabro F, Larsen B, Tervo-Clemmens B, Elliot S, Foran W, … Luna B (2021). Dopamine-related striatal neurophysiology is associated with specialization of frontostriatal reward circuitry through adolescence. Progress in Neurobiology, 201, 101997. doi: 10.1016/j.pneurobio.2021.101997 33667595 PMC8096717

[R66] Pizzagalli DA (2014). Depression, stress, and anhedonia: Toward a synthesis and integrated model. Annual of Clinical Psychology, 10, 393–423. doi: 10.1146/annurev-clinpsy-050212-185606 PMC3972338 24471371

[R67] Platt B, Kadosh KC, & Lau JY (2013). The role of peer rejection in adolescent depression. Depression and Anxiety, 30(9), 809–821. doi: 10.1002/da.22120 23596129

[R68] Poznanski EO, & Mokros HB (1996). Children’s depression rating scale, revised (CDRS-R). Los Angeles: Western Psychological Services.

[R69] R Core Team (2020). R: A language and environment for statistical computing. [Computer software]. R Foundation for Statistical Computing. https://www.R-project.org/.

[R70] Reynolds WM (2010). Reynolds adolescent depression scale. Atlanta, GA, USA: The Corsini Encyclopedia of Psychology, 1–1. doi: 10.1002/9780470479216.corpsy0798

[R71] Roozendaal B, McEwen BS, & Chattarji S (2009). Stress, memory and the amygdala. Nature Reviews Neuroscience, 10(6), 423–433. doi: 10.1038/nrn2651 19469026

[R72] Rosso IM, Cintron CM, Steingard RJ, Renshaw PF, Young AD, & Yurgelun-Todd DA (2005). Amygdala and hippocampus volumes in pediatric major depression. Biological Psychiatry, 57(1), 21–26. doi: 10.1016/j.biopsych.2004.10.027 15607296

[R73] Rueger SY, Malecki CK, Pyun Y, Aycock C, & Coyle S (2016). A meta-analytic review of the association between perceived social support and depression in childhood and adolescence. Psychological Bulletin, 142(10), 1017–1067. doi: 10.1037/bul0000058 27504934

[R74] Salamone J, Correa M, Mingote S, & Weber S (2005). Beyond the reward hypothesis: Alternative functions of nucleus accumbens dopamine. Current Opinion in Pharmacology, 5(1), 34–41. doi: 10.1016/j.coph.2004.09.004 15661623

[R75] Saleh K, Carballedo A, Lisiecka D, Fagan AJ, Connolly G, Boyle G, & Frodl T (2012). Impact of family history and depression on amygdala volume. Psychiatry Research: Neuroimaging, 203(1), 24–30. 10.1016/j.pscychresns.2011.10.004 22867951

[R76] Schmaal L (2019). Cortical surface area: a potential biological marker for depression onset and poor clinical outcomes. The Lancet Psychiatry, 6(4), 277–279. 30904115 10.1016/S2215-0366(19)30100-2

[R77] Schmaal L, Yücel M, Ellis R, Vijayakumar N, Simmons JG, Allen NB, & Whittle S (2017). Brain structural signatures of adolescent depressive symptom trajectories: A longitudinal magnetic resonance imaging study. Journal of the American Academy of Child & Adolescent Psychiatry, 56(7), 593–601. doi: 10.1016/j.pscychresns.2011.10.004 28647011

[R78] Sheets ES, & Craighead WE (2014). Comparing chronic interpersonal and noninterpersonal stress domains as predictors of depression recurrence in emerging adults. Behaviour Research and Therapy, 63, 36–42. doi: 10.1016/j.brat.2014.09.001 25277497 PMC4258528

[R79] Sheline YI, Liston C, & McEwen BS (2019). Parsing the hippocampus in depression: Chronic stress, hippocampal volume, and major depressive disorder. Biological Psychiatry, 85(6), 436–438. doi: 10.1016/j.biopsych.2019.01.011 30777168

[R80] Shenhav A, Botvinick MM, & Cohen JD (2013). The expected value of control: an integrative theory of anterior cingulate cortex function. Neuron, 79(2), 217–240. 23889930 10.1016/j.neuron.2013.07.007 PMC3767969

[R81] Sheridan MA, & McLaughlin KA (2020). Neurodevelopmental mechanisms linking ACEs with psychopathology. In Asmundson JGG & Afifi TO (Eds.), Adverse childhood experiences (pp. 265–285). Cambridge, MA, USA: Academic Press. doi: 10.1016/B978-0-12-816065-7.00013-6

[R82] Siegle GJ, Steinhauer SR, Thase ME, Stenger VA, & Carter CS (2002). Can’t shake that feeling: Event-related fMRI assessment of sustained amygdala activity in response to emotional information in depressed individuals. Biological Psychiatry, 51(9), 693–707. doi: 10.1016/s0006-3223(02)01314-8 11983183

[R83] Slavich GM (2019). Stressnology: The primitive (and problematic) study of life stress exposure and pressing need for better measurement. Brain, Behavior, and Immunity, 75, 3–5. doi: 10.1016/j.bbi.2018.08.011 30236597 PMC6279572

[R84] Slavich GM, & Irwin MR (2014). From stress to inflammation and major depressive disorder: A social signal transduction theory of depression. Psychological Bulletin, 140(3), 774–815. doi: 10.1037/a0035302 24417575 PMC4006295

[R85] Slavich GM, O’Donovan A, Epel ES, & Kemeny ME (2010a). Black sheep get the blues: A psychobiological model of social rejection and depression. Neuroscience & Biobehavioral Reviews, 35(1), 39–45. doi: 10.1016/j.neubiorev.2010.01.003 20083138 PMC2926175

[R86] Slavich GM, & Sacher J (2019). Stress, sex hormones, inflammation, and major depressive disorder: Extending social signal transduction theory of depression to account for sex differences in mood disorders. Psychopharmacology, 236(10), 3063–3079. doi: 10.1007/s00213-019-05326-9 31359117 PMC6821593

[R87] Slavich GM, & Shields GS (2018). Assessing lifetime stress exposure using the Stress and Adversity Inventory for Adults (Adult STRAIN): An overview and initial validation. Psychosomatic Medicine, 80(1), 17–27. doi: 10.1097/PSY.0000000000000534 29016550 PMC5757659

[R88] Slavich GM, Stewart JG, Esposito EC, Shields GS, & Auerbach RP (2019). The Stress and Adversity Inventory for Adolescents (Adolescent STRAIN): Associations with mental and physical health, risky behaviors, and psychiatric diagnoses in youth seeking treatment. Journal of Child Psychology and Psychiatry, 60(9), 998–1009. doi: 10.1111/jcpp.13038 30912589 PMC6692180

[R89] Slavich GM, Way BM, Eisenberger NI, & Taylor SE (2010b). Neural sensitivity to social rejection is associated with inflammatory responses to social stress. Proceedings of the National Academy of Sciences of the USA, 107(33), 14817–14822. doi: 10.1073/pnas.1009164107 20679216 PMC2930449

[R90] Somerville LH, Jones RM, & Casey BJ (2010). A time of change: Behavioral and neural correlates of adolescent sensitivity to appetitive and aversive environmental cues. Brain Cognition, 72(1), 124–133. doi: 10.1016/j.bandc.2009.07.003 19695759 PMC2814936

[R91] Steinberg L (2005). Cognitive and affective development in adolescence. Trends in Cognitive Science, 9(2), 69–74. doi: 10.1016/j.tics.2004.12.005 15668099

[R92] Storsve AB, Fjell AM, Tamnes CK, Westlye LT, Overbye K, Aasland HW, & Walhovd KB (2014). Differential longitudinal changes in cortical thickness, surface area and volume across the adult life span: regions of accelerating and decelerating change. Journal of Neuroscience, 34(25), 8488–8498. 24948804 10.1523/JNEUROSCI.0391-14.2014 PMC6608217

[R93] Sturmbauer SC, Shields GS, Hetzel EL, Rohleder N, & Slavich GM (2019). The Stress and Adversity Inventory for Adults (Adult STRAIN) in German: An overview and initial validation. PLoS ONE, 14(5), e0216419. 31071135 10.1371/journal.pone.0216419 PMC6508721

[R94] Teicher MH, Samson JA, Anderson CM, & Ohashi K (2016). The effects of childhood maltreatment on brain structure, function and connectivity. Nature Reviews Neuroscience, 17(10), 652–666. doi: 10.1038/nrn.2016.111 27640984

[R95] Tottenham N, & Galván A (2016). Stress and the adolescent brain: Amygdala-prefrontal cortex circuitry and ventral striatum as developmental targets. Neuroscience & Biobehavioral Reviews, 70, 217–227. doi: 10.1016/j.neubiorev.2016.07.030 27473936 PMC5074883

[R96] Twenge JM, Cooper AB, Joiner TE, Duffy ME, & Binau SG (2019). Age, period, and cohort trends in mood disorder indicators and suicide-related outcomes in a nationally representative dataset, 2005–2017. Journal of Abnormal Psychology, 128(3), 185. doi: 10.1037/abn0000410 30869927

[R97] van Harmelen AL, Blakemore SJ, Goodyer IM, & Kievit RA (2021). The interplay between adolescent friendship quality and resilient functioning following childhood and adolescent adversity. Adversity and Resilience Science, 2(1), 37–50. doi: 10.1007/s42844-020-00027-1 PMC7615274 37915317

[R98] van Harmelen AL, Gibson JL, St Clair MC, Owens M, Brodbeck J, Dunn V, … Goodyer IM (2016). Friendships and family support reduce subsequent depressive symptoms in at-risk adolescents. PLoS ONE, 11(5), e0153715. doi: 10.1371/journal.pone.0153715 27144447 PMC4856353

[R99] van Harmelen AL, Kievit RA, Ioannidis K, Neufeld S, Jones PB, Bullmore E, … Consortium NSPN (2017). Adolescent friendships predict later resilient functioning across psychosocial domains in a healthy community cohort. Psychological Medicine, 47(13), 2312–2322. doi: 10.1017/S0033291717000836 28397612 PMC5820532

[R100] van Marle HJF, Hermans EJ, Qin S, & Fernández G (2009). From specificity to sensitivity: How acute stress affects amygdala processing of biologically salient stimuli. Biological Psychiatry, 66(7), 649–655. doi: 10.1016/j.biopsych.2009.05.014 19596123

[R101] Vrshek-Schallhorn S, Stroud CB, Mineka S, Hammen C, Zinbarg RE, Wolitzky-Taylor K, & Craske MG (2015). Chronic and episodic interpersonal stress as statistically unique predictors of depression in two samples of emerging adults. Journal of Abnormal Psychology, 124(4), 918. doi: 10.1037/abn0000088 26301973 PMC4948584

[R102] Wacker J, Dillon DG, & Pizzagalli DA (2009). The role of the nucleus accumbens and rostral anterior cingulate cortex in anhedonia: Integration of resting EEG, fMRI, and volumetric techniques. NeuroImage, 46(1), 327–337. doi: 10.1016/j.neuroimage.2009.01.058 19457367 PMC2686061

[R103] Wager TD, van Ast VA, Hughes BL, Davidson ML, Lindquist MA, & Ochsner KN (2009). Brain mediators of cardiovascular responses to social threat, part II: Prefrontal-subcortical pathways and relationship with anxiety. NeuroImage, 47(3), 836–851. doi: 10.1016/j.neuroimage.2009.05.044 19465135 PMC4169880

[R104] Walker JC, Teresi GI, Weisenburger RL, Segarra JR, Ojha A, Kulla A, … Ho TC (2020). Study protocol for teen inflammation glutamate emotion research (TIGER). Frontiers in Human Neuroscience, 14, 585512. doi: 10.3389/fnhum.2020.585512 33192421 PMC7604389

[R105] Wang H, Braun C, & Enck P (2017). How the brain reacts to social stress (exclusion) – a scoping review. Neuroscience & Biobehavioral Reviews, 80, 80–88. doi: 10.1016/j.neubiorev.2017.05.012 28535967

[R106] Webb CA, Olson EA, Killgore WD, Pizzagalli DA, Rauch SL, & Rosso IM (2018). Rostral anterior cingulate cortex morphology predicts treatment response to internet-based cognitive behavioral therapy for depression. Biological Psychiatry: Cognitive Neuroscience and Neuroimaging, 3(3), 255–262. doi: 10.1016/j.bpsc.2017.08.005 29486867 PMC6005352

[R107] Wei C, Hoff A, Villabø MA, Peterman J, Kendall PC, Piacentini J … March J (2014). Assessing anxiety in youth with the multidimensional anxiety scale for children. Journal of Clinical Child & Adolescent Psychology, 43, 566–578. doi: 10.1080/15374416.2013.814541 23845036 PMC3858516

[R108] Whittle S, Lichter R, Dennison M, Vijayakumar N, Schwartz O, Byrne ML, … Allen NB (2014). Structural brain development and depression onset during adolescence: A prospective longitudinal study. American Journal of Psychiatry, 171(5), 564–571. doi: 10.1176/appi.ajp.2013.13070920 24577365

[R109] Wierenga LM, Langen M, Oranje B, & Durston S (2014). Unique developmental trajectories of cortical thickness and surface area. Neuroimage, 87, 120–126. 24246495 10.1016/j.neuroimage.2013.11.010

[R110] World Health Organization. (2017). Depression and other common mental disorders: global health estimates. Retrieved from https://apps.who.int/iris/bitstream/handle/10665/254610/W?sequence=1.

[R111] Zhang Y, Yang Y, Zhu L, Zhu Q, Jia Y, Zhang L, … Wang J (2021). Volumetric deficit within the fronto-limbic-striatal circuit in first-episode drug naïve patients with major depression disorder. Frontiers in Psychiatry, 11, 600583. doi: 10.3389/fpsyt.2020.600583 33551870 PMC7854541

[R112] Zisook S, Rush AJ, Lesser I, Wisniewski SR, Trivedi M, Husain MM, … Fava M (2007). Preadult onset vs. adult onset of major depressive disorder: A replication study. Acta Psychiatrica Scandinavica, 115(3), 196–205. doi: 10.1111/j.1600-0447.2006.00868.x 17302619

