# Supplementary material for: Social threat, fronto-cingulate-limbic morphometry, and symptom course in depressed adolescents: a longitudinal investigation
Source: Psychol Med. 2022 Sep 19;53(11):5203–17. doi: 10.1017/S0033291722002239 (PMC10024647; doi:10.1017/S0033291722002239)
Supplement: Supplementary file 1 [file S0033291722002239sup001.docx]

**Supplementary Material for**

**“Social threat, fronto-cingulate-limbic morphometry, and symptom course in adolescents with depression: a longitudinal investigation”**

**Supplementary Methods**

*Participants*

Adolescent participants (<18 years old) and their parent/legal guardian provided written assent and informed consent, respectively. Adult participants (≥18 years old) provided written informed consent. All participants were financially compensated for their participation.

If a participant met subthreshold criteria for a depressive disorder based on the K-SADS-PL, they were also required to have a summary score above the clinical threshold on the Children’s Depressive Rating Scale–Revised (CDRS-R; see *Clinical assessments*, below).

Exclusion criteria were premenarcheal status (for females); current developmental or intellectual disorders that would interfere with the ability to comprehend study procedures; contraindications to MRI (e.g., claustrophobia, presence of ferromagnetic metal in or on body); concussion within the past 6 weeks or lifetime concussion with loss of consciousness; lifetime or current diagnosis of DSM-IV Bipolar Disorder, symptoms of psychosis, or Alcohol Dependence; or current diagnosis of DSM-5 Moderate Substance Use Disorder with substance-specific withdrawal.

*Procedure*

Participants were recruited using flyers, advertisements on Craigslist, Nextdoor, and Facebook, and an internal referral program. Before being invited for a laboratory visit to confirm eligibility, adolescents and their parent/legal guardian completed a voluntary screening interview over the phone where they responded to questions about the adolescent’s current symptoms of depression, lifetime history of mania and symptoms of psychosis, current substance use, and other exclusion/inclusion criteria.

*Clinical assessments*

The K-SADS-PL is a structured diagnostic clinical interview used to generate valid and reliable DSM-IV Axis I disorder diagnoses in children. Participants and their parent/legal guardian were also administered the CDRS-R to yield a dimensional measure of depression severity.

The CDRS-R is a 17-item clinician-rated semi-structured interview that has demonstrated strong validity and reliability in adolescents ([Mayes et al., 2010](https://www-sciencedirect-com.stanford.idm.oclc.org/science/article/pii/S0165032720327415?via%3Dihub#bib0039)). Interviewers assigned a total summary score to each participant by integrating parent and adolescent responses. Participants who were subthreshold for a depressive disorder based on the K-SADS-PL were also required to have a raw summary score $\geq$ 30 (equivalent to a *t*-score $\geq$ 55). All diagnoses and CDRS-R summary scores were reviewed for accuracy and reliability by a clinically trained research team.

*Life stress*

We assessed the severity, frequency, timing, and duration of 75 different stressors—including 33 acute life events and 42 chronic difficulties—across 12 primary life domains (i.e., Housing, Education, Work, Treatment/Health, Marital/Partner, Reproduction, Financial, Legal/Crime, Other Relationships, Parent/Guardian, Death, Life-Threatening Situations) and five social-psychological characteristics (i.e., Interpersonal Loss, Physical Danger, Humiliation, Entrapment, Role Change/Disruption) (Slavich et al., 2019).

Five participants with usable structural MRI data completed the Adolescent STRAIN outside of the 3-week administration interval (mean days for these 5 participants following T1V1=69.4). These 5 participants did not differ in their reports of social stressor severity from the 52 who completed the Adolescent STRAIN within the 3 weeks (*p*=0.91, Hedges’s *g*=-0.019). Thus, we included these 5 participants in our final analyses.

*MRI scanning acquisition*

All participants completed a T1-weighted anatomical scan obtained using a spoiled gradient (SPGR) sequence (TR/TE/TI = 8.2/3.2/600 ms; flip angle = 12°; 156 axial slices; FOV = 25.6 cm; matrix = 256 mm x 256 mm, isotropic voxel = 1 mm, total scan time: 3:40). GMV estimates of the amygdala, hippocampus, and rostral/caudal ACC were generated using the *recon-all* function from FreeSurfer v. 6.0 (Fischl et al., 2012).

*MRI quality control*

Our quality control procedures for examining the anatomical MRI scans included the following: 1) visually inspecting each T1-weighted image to determine usability based on motion (i.e., “ringing” artifacts) prior to segmentation; 2) visually inspecting each automated segmentation output from FreeSurfer against the T1-weighted volume for accuracy; and 3) extracting volumes for each hemisphere and converted them to z-scores and visually examining any segmentations for volumes where z-scores ≥ |2.5|. Any segmentations (left or right, separately) that failed any of these steps were conservatively removed from final analyses.

*Exploratory whole-brain analyses*

We used the *QDECR* package in R (Lamballais & Muetzal, 2021) to perform vertex-wise surface-based whole-brain analyses on cortical areas, volumes, and thickness. Specifically, we ran linear regressions to test associations between total lifetime social threat severity and cortical morphology while controlling for age, antidepressant usage, and session type (i.e., pre/post COVID-19). Results were corrected for multiple comparisons using pre-cached smoothed Gaussian Monte Carlo on clusters (*p*=0.001).

**Supplementary Results**

*Sensitivity analyses (lateralization)*

Regarding subcortical structures, *post-hoc* lateralization analyses revealed that higher reported lifetime social threat severity was associated with smaller GMVs in both the right (β=-0.39, *p*_FDR_=0.016) and left amygdala (β=-0.35, *p*_FDR_=0.023). Higher reported lifetime social threat severity was also associated with smaller right NAcc GMV (β=-0.47, *p*_FDR_=0.003) but not with left NAcc GMV (*p*_FDR_=0.172).

Regarding cortical regions, *post-hoc* lateralization analyses revealed that lifetime social threat severity was not associated with either right or left cACC surface areas (left, *p*_FDR_=0.155; right, *p*_FDR_=0.082), or with either right of left rACC surface areas (left, *p*_FDR_=0.088; right, *p*_FDR_=0.082).

Specificity analyses testing lateralization of the findings for predicting RADS-2 scores longitudinally revealed that smaller right rACC and cACC surface areas and left amygdala and NAcc GMVs were associated with greater depression symptoms over 9 months (all βs≤-0.26, *p*_FDR_≤ 0.042); left rACC and cACC surface areas and right amygdala and NAcc GMVs were not associated with depression symptoms over 9 months (all *p*_FDR_≥0.075).

**Supplementary Tables**

**Table S1A.** **Fixed effects estimated from linear models testing associations between lifetime social threat and non-social threat severity with RADS-2 at baseline with sex as a covariate.** All statistical models included age and antidepressant usage as covariates. β refers to the standardized partial regression coefficients. CI=confidence interval; df=degrees of freedom; SE=standard error **p*<0.05, ***p*<0.01, ****p*<0.001.

|  | *β* | *SE* | *95% CI* | *t (df)* | *Uncorrected p* |
| --- | --- | --- | --- | --- | --- |
| Lifetime social threat severity | 0.35 | 0.14 | [0.07, 0.63] | 2.51 (51) | 0.015* |
| Lifetime non-social threat severity | 0.40 | 0.14 | [0.12, 0.67] | 2.91 (51) | 0.005** |

**Table S1B. Fixed effects estimated from linear models testing associations between lifetime social threat and non-social threat severity with RADS-2 longitudinally with sex as a covariate.** All statistical models included age, antidepressant usage (as a time-varying factor), time, and random effects of intercept and slope as covariates. β refers to the standardized partial regression coefficients. CI=confidence interval; df=degrees of freedom; SE=standard error **p*<0.05, ***p*<0.01, ****p*<0.001.

|  | *β* | *SE* | *95% CI* | *t (df)* | *Uncorrected p* |
| --- | --- | --- | --- | --- | --- |
| Lifetime social threat severity | 0.22 | 0.12 | [-0.01, 0.45] | 1.89 (53.22) | 0.065 |
| Lifetime non-social threat severity | 0.23 | 0.12 | [-0.01, 0.46] | 1.90 (53.63) | 0.062 |

**Table S1C. Fixed effects estimated from linear models testing associations between lifetime social threat severity and brain morphometry at baseline with sex as a covariate.** All statistical models included age, antidepressant usage, and session type as covariates. β refers to the standardized partial regression coefficients. cACC=caudal anterior cingulate cortex; CI=confidence interval; df=degrees of freedom; GMV=gray matter volumes; NAcc=nucleus accumbens; rACC=rostral anterior cingulate cortex; SE=standard error **p*<0.05, ***p*<0.01, ****p*<0.001.

|  | *β* | *SE* | *95% CI* | *t (df)* | *Uncorrected p* |
| --- | --- | --- | --- | --- | --- |
| rACC surface area | -0.24 | 0.14 | [-0.52, 0.04] | -1.71 (51) | 0.093 |
| cACC surface area | -0.26 | 0.14 | [-0.55, 0.03] | -1.82 (51) | 0.074 |
| NAcc GMV | -0.30 | 0.14 | [-0.59, -0.02] | -2.15 (51) | 0.036* |
| Amygdala GMV | -0.29 | 0.14 | [-0.57, -0.02] | -2.13 (51) | 0.038* |

**Table S1D. Fixed effects estimated from linear models testing associations between brain morphology and lifetime non-social stress severity at baseline with sex as a covariate.** All statistical models included age, antidepressant usage, and session type as covariates. β refers to the standardized partial regression coefficients. cACC=caudal anterior cingulate cortex; CI=confidence interval; df=degrees of freedom; GMV=gray matter volumes; NAcc=nucleus accumbens; rACC=rostral anterior cingulate cortex; SE=standard error **p*<0.05, ***p*<0.01, ****p*<0.001.

|  | *β* | *SE* | *95% CI* | *t (df)* | *Uncorrected p* |
| --- | --- | --- | --- | --- | --- |
| Amygdala GMV | -0.23 | 0.14 | [-0.15, 0.05] | -1.63 | 0.109 |
| cACC surface area | -0.18 | 0.14 | [-0.47, 0.11] | -1.21 (51) | 0.230 |
| NAcc GMV | -0.14 | 0.15 | [-0.43, 0.16] | -0.94 | 0.354 |
| rACC surface area | -0.06 | 0.14 | [-0.35, 0.23] | -0.40 (51) | 0.688 |

**Table S1E. Fixed effects estimated from linear models testing associations between brain morphology and RADS-2 at baseline with sex as a covariate.** All statistical models included age, antidepressant usage, and session type as covariates. β refers to the standardized partial regression coefficients. cACC=caudal anterior cingulate cortex; CI=confidence interval; df=degrees of freedom; GMV=gray matter volumes; NAcc=nucleus accumbens; rACC=rostral anterior cingulate cortex; SE=standard error **p*<0.05, ***p*<0.01, ****p*<0.001.

|  | *β* | *SE* | *95% CI* | *t (df)* | *Uncorrected p* |
| --- | --- | --- | --- | --- | --- |
| Amygdala GMV | -0.34 | 0.12 | [-0.58, -0.10] | -2.83 (57) | 0.006** |
| cACC surface area | -0.31 | 0.13 | [-0.56, -0.06] | -2.49 (57) | 0.016* |
| NAcc GMV | -0.37 | 0.12 | [-0.62, -0.13] | -3.07 (57) | 0.003** |
| rACC surface area | -0.30 | 0.12 | [-0.55, -0.05] | -2.43 (57) | 0.018* |

**Table S1F. Fixed effects estimated from linear models testing associations between brain morphology and RADS-2 longitudinally with sex as a covariate.** All statistical models included age, antidepressant usage (as a time-varying factor), session type, time, and random effects of intercept and slope as covariates. β refers to the standardized partial regression coefficients. cACC=caudal anterior cingulate cortex; CI=confidence interval; df=degrees of freedom; GMV=gray matter volumes; NAcc=nucleus accumbens; rACC=rostral anterior cingulate cortex; SE=standard error **p*<0.05, ***p*<0.01, ****p*<0.001.

|  | *β* | *SE* | *95% CI* | *t (df)* | *Uncorrected p* |
| --- | --- | --- | --- | --- | --- |
| Amygdala GMV | -0.23 | 0.12 | [-0.46, 0.01] | -1.91 (52.23) | 0.061 |
| cACC surface area | -0.23 | 0.11 | [-0.46, -0.01] | -2.05 (50.75) | 0.045* |
| NAcc GMV | -0.24 | 0.11 | [-0.47, -0.02] | -2.14 (53.87) | 0.037* |
| rACC surface area | -0.22 | 0.11 | [-0.45, 0.00] | -1.96 (49.76) | 0.056 |

**Table S2A. Model fit comparisons of primary cross-sectional models examining predictors of RADS-2 cross-sectionally outcomes in association with lifetime social threat severity with and without sex as a covariate****.** All statistical models included age, antidepressant usage, and session type (where appropriate) as covariates. The *p­-*value in the last column indicates if there is a significant difference in model fit. AIC=Akaike Information Criteria; cACC=caudal anterior cingulate cortex; GMV=gray matter volumes; NAcc=nucleus accumbens; rACC=rostral anterior cingulate cortex. **p*<0.05, ***p*<0.01, ****p*<0.001.

| Predictor | AIC with sex covariate | AIC without sex covariate | *P* |
| --- | --- | --- | --- |
| Lifetime social stress severity | 447.75 | 447.34 | 0.234 |
| Lifetime non-social stress severity | 445.67 | 445.74 | 0.175 |

**Table S2B. Model fit comparisons of primary models examining predictors of RADS-2 scores longitudinally.** All statistical models included age, antidepressant usage (as a time-varying factor), session type (where appropriate), time, and random effects of intercept and slope as covariates. The *p­-*value in the last column indicates if there is a significant difference in model fit. AIC=Akaike Information Criteria; cACC=caudal anterior cingulate cortex; GMV=gray matter volumes; NAcc=nucleus accumbens; rACC=rostral anterior cingulate cortex. **p*<0.05, ***p*<0.01, ****p*<0.001.

| Predictor | AIC with sex covariate | AIC without sex covariate | *P* |
| --- | --- | --- | --- |
| Lifetime social threat severity | 508.30 | 509.04 | 0.098 |
| Lifetime non-social threat severity | 508.56 | 510.08 | 0.054 |
| Amygdala GMV | 509.87 | 509.49 | 0.202 |
| NAcc GMV | 508.78 | 509.24 | 0.117 |
| cACC surface area | 509.23 | 509.22 | 0.159 |
| rACC surface area | 509.54 | 509.38 | 0.175 |

**Table S2C. Model fit comparisons of primary cross-sectional models examining associations between lifetime social threat severity and brain morphometry with and without sex as a covariate.** All statistical models included age, antidepressant usage, and session type as covariates. The *p­-*value in the last column indicates if there is a significant difference in model fit. AIC=Akaike Information Criteria; cACC=caudal anterior cingulate cortex; GMV=gray matter volumes; NAcc=nucleus accumbens; rACC=rostral anterior cingulate cortex. **p*<0.05, ***p*<0.01, ****p*<0.001.

| Brain outcome measure | AIC with sex covariate | AIC without sex covariate | *P* |
| --- | --- | --- | --- |
| Amygdala GMV | 749.06 | 754.40 | 0.011* |
| NAcc GMV | 655.35 | 655.17 | 0.205 |
| cACC surface area | 692.26 | 694.97 | 0.041* |
| cACC cortical thickness | -9.13 | -10.80 | 0.593 |
| rACC surface area | 697.20 | 700.92 | 0.024* |
| rACC cortical thickness | -35.92 | -36.57 | 0.275 |

**Table S2D. Model fit comparisons of primary cross-sectional models examining associations between lifetime non-social threat severity and brain morphometry with and without sex as a covariate.** All statistical models included age, antidepressant usage, and session type as covariates. The *p­-*value in the last column indicates if there is a significant difference in model fit. AIC=Akaike Information Criteria; cACC=caudal anterior cingulate cortex; GMV=gray matter volumes; NAcc=nucleus accumbens; rACC=rostral anterior cingulate cortex. **p*<0.05, ***p*<0.01, ****p*<0.001.

| Brain outcome measure | AIC with sex covariate | AIC without sex covariate | *P* |
| --- | --- | --- | --- |
| Amygdala GMV | 751.00 | 757.71 | 0.005** |
| NAcc GMV | 659.33 | 660.29 | 0.106 |
| cACC surface area | 694.24 | 698.09 | 0.023* |
| cACC cortical thickness | -13.23 | -15.08 | 0.716 |
| rACC surface area | 700.21 | 705.70 | 0.001* |
| rACC cortical thickness | -39.32 | -39.51 | 0.206 |

**Table S3A. Fixed effects estimated from linear models testing associations between lifetime social threat and non-social threat with MASC-2 at baseline.** All statistical models included age and antidepressant usage as covariates. β refers to the standardized partial regression coefficients. CI=confidence interval; df=degrees of freedom; SE=standard error **p*<0.05, ***p*<0.01, ****p*<0.001.

|  | *β* | *SE* | *95% CI* | *t (df)* | *Uncorrected p* |
| --- | --- | --- | --- | --- | --- |
| Lifetime social threat severity | 0.43 | 0.13 | [0.16, 0.69] | 3.25 (52) | 0.002** |
| Lifetime non-social threat severity | 0.17 | 0.14 | [-0.12, 0.46] | 1.21 (52) | 0.233 |

**Table S3B. Fixed effects estimated from linear models testing associations between lifetime social threat and non-social threat severity and MASC-2 longitudinally.** All statistical models included age, antidepressant usage (as a time-varying factor), session type (where appropriate), time, and random effects of intercept and slope as covariates. β refers to the standardized partial regression coefficients. CI=confidence interval; df=degrees of freedom; SE=standard error **p*<0.05, ***p*<0.01, ****p*<0.001.

|  | *β* | *SE* | *95% CI* | *t (df)* | *Uncorrected p* |
| --- | --- | --- | --- | --- | --- |
| Lifetime social threat severity | 0.37 | 0.12 | [ 0.14,  0.60] | 3.19 (52.68) | 0.002** |
| Lifetime non-social threat severity | 0.16 | 0.13 | [-0.09, 0.42] | 1.26 (53.61) | 0.214 |

**Table S3C. Fixed effects estimated from linear models testing for associations between brain morphology and MASC-2 at baseline.** All statistical models included age, antidepressant usage, and session type as covariates. β refers to the standardized partial regression coefficients. cACC=caudal anterior cingulate cortex; CI=confidence interval; df=degrees of freedom; GMV=gray matter volumes; L=left; NAcc=nucleus accumbens; R=right; rACC=rostral anterior cingulate cortex; SE=standard error **p*<0.05, ***p*<0.01, ****p*<0.001.

|  | *β* | *SE* | *95% CI* | *t (df)* | *Uncorrected p* |
| --- | --- | --- | --- | --- | --- |
| Amygdala GMV | -0.27 | 0.12 | [-0.51, -0.02] | -2.19 (58) | 0.033* |
| cACC surface area | -0.24 | 0.12 | [-0.49, 0.00] | -1.97 (58) | 0.054 |
| NAcc GMV | -0.39 | 0.12 | [-0.63, -0.14] | -3.18 (58) | 0.002** |
| rACC surface area | -0.21 | 0.13 | [-0.46, 0.04] | -1.66 (58) | 0.101 |

**Table S3D. Fixed effects estimated from linear models testing associations between brain morphology and MASC-2 longitudinally.** All statistical models included age, antidepressant usage (as a time-varying factor), session type (where appropriate), time, and random effects of intercept and slope as covariates. β refers to the standardized partial regression coefficients. cACC=caudal anterior cingulate cortex; CI=confidence interval; df=degrees of freedom; GMV=gray matter volumes; L=left; NAcc=nucleus accumbens; R=right; rACC=rostral anterior cingulate cortex; SE=standard error **p*<0.05, ***p*<0.01, ****p*<0.001.

|  | *β* | *SE* | *95% CI* | *t (df)* | *Uncorrected p* |
| --- | --- | --- | --- | --- | --- |
| Amygdala GMV | -0.28 | 0.12 | [-0.51, -0.05] | -2.37 (52.92) | 0.022* |
| cACC surface area | -0.25 | 0.12 | [-0.48, -0.01] | -2.07 (51.50) | 0.044* |
| NAcc GMV | -0.36 | 0.12 | [-0.59, -0.13] | -3.12 (53.62) | 0.003** |
| rACC surface area | -0.23 | 0.12 | [-0.46, 0.01] | -1.89 (51.45) | 0.065 |

**Table S3E. Indirect effects estimated from mediation models testing if brain morphometry statistically mediated the association between lifetime social threat severity and MASC-2 longitudinally.** All statistical models included age, antidepressant usage, and session type (where appropriate) as covariates. Refer to the Methods for a detailed description of how the mediation models were constructed. β refers to the standardized partial regression coefficients. CI=confidence interval; SE=standard error.

|  | *β* | *SE* | *95% CI* |
| --- | --- | --- | --- |
| Amygdala GMV | 0.11 | 0.06 | [0.01, 0.25] |
| cACC surface area | 0.08 | 0.16 | [-0.001, 0.21] |
| NAcc GMV | 0.13 | 0.07 | [0.02, 0.28] |
| rACC surface area | 0.07 | 0.05 | [-0.01, 0.20] |

**Table S4. Results from whole-brain vertex-wise analyses showing significant effects of social threat on surface area and cortical volumes.** All regions are described based on spatial correspondence with labels from the Automated Anatomical Labeling (AAL) atlas. Hemi=hemisphere; LH=left hemisphere; NA=not applicable; RH=right hemisphere; SE=standard error.

| Morphometry variable | Hemi | # of vertices | Mean coefficient | Mean SE | Top region 1 | Top region 2 | Top region 3 |
| --- | --- | --- | --- | --- | --- | --- | --- |
| area | LH | 1896 | -0.003 | 0.001 | fusiform | parahippocampal | inferiortemporal |
| area | LH | 980 | -0.005 | 0.001 | inferiorparietal | superiorparietal | NA |
| area | LH | 1086 | -0.004 | 0.001 | postcentral | supramarginal | NA |
| volume | LH | 558 | -0.011 | 0.003 | inferiorparietal | superiorparietal | NA |
| volume | LH | 474 | -0.017 | 0.004 | fusiform | NA | NA |
| area | RH | 1069 | -0.005 | 0.001 | superiorfrontal | rostralmiddlefrontal | NA |

**Supplementary Figures**

**Figure S1.** Study timeline with sample size at each time point. *Intervals are presented as mean ± SD time (in years) since T1.


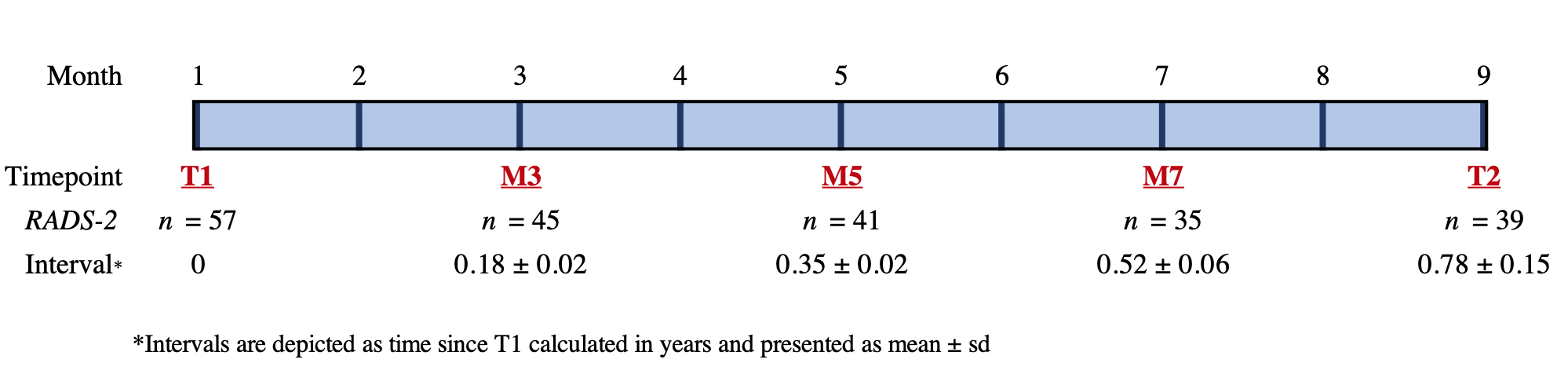


**Figure S2.** Correlation matrix of cross-sectional continuous variables of interest. All bivariate correlations were assessed using Pearson’s correlations. Shaded cells indicate significant associations (uncorrected *p*<0.05). Abbreviations: AMYG=amygdala; HPC=hippocampus; NAcc=nucleus accumbens; rACC=rostral anterior cingulate cortex; cACC=caudal anterior cingulate cortex; CT=cortical thickness; SA=surface area; ST=social threat severity (assessed via STRAIN); MDD=depression severity at Time 1 (assessed via RADS-2 total scores); ICV=intracranial volume.


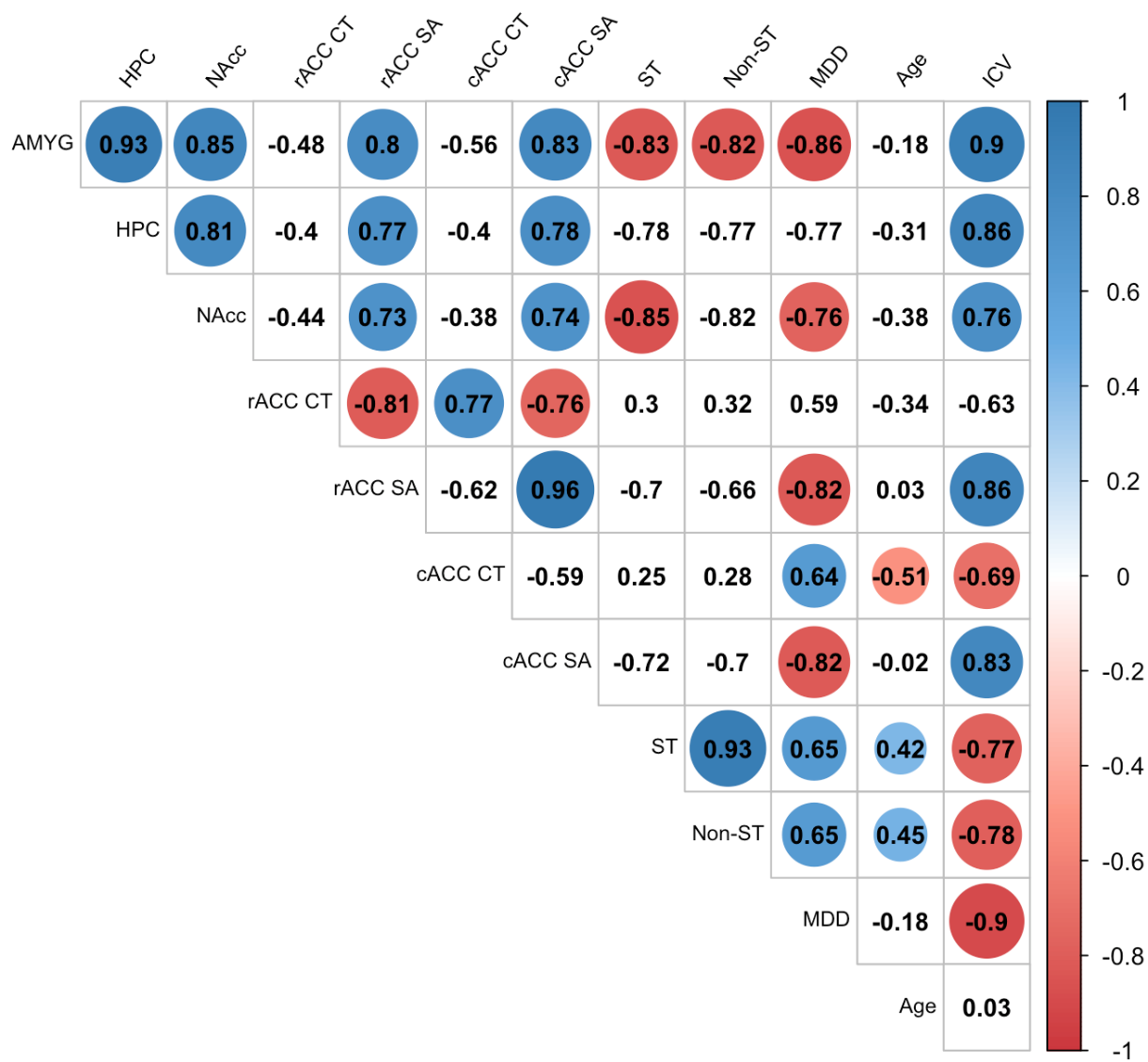


**Figure S3. Results from whole-brain vertex-wise analyses showing significant effects of social threat on surface area and cortical volumes.** A) Greater lifetime social threat severity was associated with lower cortical surface areas in left fusiform gyrus, inferior parietal regions, and postcentral gyrus. B) Greater lifetime social threat severity was associated with lower cortical volume in left fusiform gyrus and left inferior parietal regions. C) Greater lifetime social threat severity was associated with lower right superior frontal surface area. Consistent with our original analyses, cortical thickness was not associated with lifetime social threat severity. In all analyses, age, antidepressant usage, and session type were included as covariates.


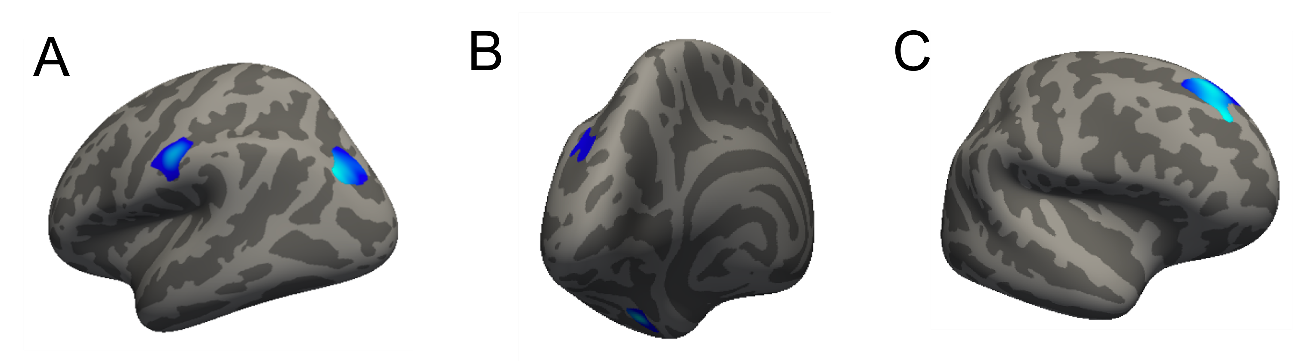


**Supplementary References**

Fischl, B. (2012). FreeSurfer. *NeuroImage*, *62*(2), 774-781. doi: [10.1016/j.neuroimage.2012.01.021](https://doi.org/10.1016/j.neuroimage.2012.01.021)

Lamballais, S., & Muetzel, R. L. (2021). QDECR: a flexible, extensible vertex-wise analysis framework in R. *Frontiers in Neuroinformatics*, *15*. doi: [10.3389/fninf.2021.561689](https://doi.org/10.3389/fninf.2021.561689)

Mayes, T. L., Bernstein, I. H., Haley, C. L., Kennard, B. D., & Emslie, G. J. (2010). Psychometric properties of the Children's Depression Rating Scale–Revised in adolescents. *Journal of Child and Adolescent Psychopharmacology*, *20*(6), 513-516. doi: [10.1089/cap.2010.0063](https://doi.org/10.1089/cap.2010.0063)

Slavich, G. M., Stewart, J. G., Esposito, E. C., Shields, G. S., & Auerbach, R. P. (2019). The Stress and Adversity Inventory for Adolescents (Adolescent STRAIN): associations with mental and physical health, risky behaviors, and psychiatric diagnoses in youth seeking treatment. *Journal of Child Psychology and Psychiatry*, *60*(9), 998-1009. doi: [10.1111/jcpp.13038](https://doi.org/10.1111/jcpp.13038)
